# Impacts on water quality in the peatland dominated catchment due to foreseen changes in Nordic Bioeconomy Pathways

**DOI:** 10.1038/s41598-023-33378-7

**Published:** 2023-04-18

**Authors:** Joy Bhattacharjee, Hannu Marttila, Eugenio Molina Navarro, Artti Juutinen, Anne Tolvanen, Arto Haara, Jouni Karhu, Bjørn Kløve

**Affiliations:** 1 Water, Energy and Environmental Engineering Research Unit, University of Oulu, PO Box 4300, 90014 Oulu, Finland; 2 Geology, Geography and Environment Department, University of Alcalá, Ctra. Madrid-Barcelona, Km. 33.6, 28805 Alcalá de Henares, Madrid Spain; 3 Natural Resources Institute Finland (LUKE), Paavo Havaksen tie 3, 90570 Oulu, Finland; 4 Natural Resources Institute Finland (LUKE), Yliopistokatu 6 B, 80100 Joensuu, Finland

**Keywords:** Biogeochemistry, Climate sciences, Environmental social sciences, Hydrology

## Abstract

The Nordic Bioeconomy Pathways (NBPs), conceptualized subsets of Shared Socioeconomic Pathways varying from environmentally friendly to open-market competition scenarios, can lead to plausible stressors in future for using bioresources. This study analysed the impacts of NBPs on hydrology and water quality based on two different land system management attributes: management strategy and a combination of reduced stand management and biomass removal at a catchment-scale projection. To understand the potential impacts of NBPs, the Simojoki catchment in northern Finland was chosen, as the catchment mainly covered peatland forestry. The analysis integrated a stakeholder-driven questionnaire, the Finnish Forest dynamics model, and Soil and Water Assessment Tool to build NBP scenarios, including Greenhouse gas emission pathways, for multiple management attributes to simulate flows, nutrients, and suspended solids (SS). For the catchment management strategy, an annual decrease in nutrients was observed for sustainability and business-as-usual scenarios. Reduced stand management and biomass removal also led to decreased export of nutrients and SS for the same scenarios, whereas, in other NBPs, the export of nutrients and SS increased with decreased evapotranspiration. Although the study was investigated at a local scale, based on the current political and socioeconomic situation, the approach used in this study can be outscaled to assess the use of forest and other bioresources in similar catchments.

## Introduction

According to the EU bioeconomy strategy, more bioresources, such as wood and crop-based biomass, are needed to move towards a low-carbon and resource-efficient society in which fossil resources are replaced by renewables^1^. In 2018 in an updated bioeconomy strategy, the emphasis was more on the sustainable management of natural resources from the land and sea^2^. Thus, the current estimate shows that the transition towards a bioresource-based economy can help to achieve sustainable development goals^3^ whereas it will increase the demand for biomass production^4,5^. Due to the unique and cross-cutting nature of bioeconomy, countries in the Nordics and Europe are adopting bioeconomy to address inter-connected challenges while having economic growth. For example, Finland has more than 30% peatland in its territory forming a substantial carbon reservoir in the boreal and subarctic regions. Globally organic soils constitute one-third of the soil carbon pool^6^. However, peatland degradation occurs due to the drainage activities^7^, modifying the water-holding capacity, water storage and also decomposition of soil layers^8^. Overall, the influence of the new and old drains is notable to hydrological conditions and nutrient loading^9–11^. However, peatland forestry produces 25% of the annual forest growth in Finland^12^. Thus, trade-offs are unavoidable as the increased need for biomass has adverse effects on natural resources^13^.

In countries where the economy relies heavily on peatland forestry or similar landuses, an increase in wood production will undoubtedly intensify the use of bioresources. This raises concerns about the transitional influence on local hydrology and water quality^14^, especially in regions with high coverage of drained peatlands. Compared to pristine peatlands, evidence of increased carbon, nutrients, and suspended solids (SS) export to watercourses in organic soils such as drained peatlands, is already well established^15^. A considerable amount of exports can occur during initial drainage, ditch network maintenance operations, and especially after the final harvest^16^. In Finland, nutrient budgets and land use impacts have been discussed over the past few years^17,18^. The analysis in those studies also indicated the effect of past peatland drainage on increased nutrient export. Even for future projections with climate input, the old drained areas (age and proportion) may have a legacy of nutrient export^19^. However, there is evidence of temporary nutrient exports^20–23^, whereas results concerning the management strategy and long-term effects of peatland forestry still require further attention^24,25^. Especially at the catchment scale, uncertainty in in-field and in-stream nutrient cycling may occur because of different management issues. Thus, balancing nutrients is a prerequisite for ecosystem sustainability^26,27^. Moreover, these processes can be more influenced by changes in the global climate^28^. Thus, the dilemma between sustainable transition and addressing the effective use of bioresources at a catchment raises issues regarding the application of possible bioeconomic pathways that can reconcile the various drivers of environmental, economic, and societal development.

**Figure 1:**
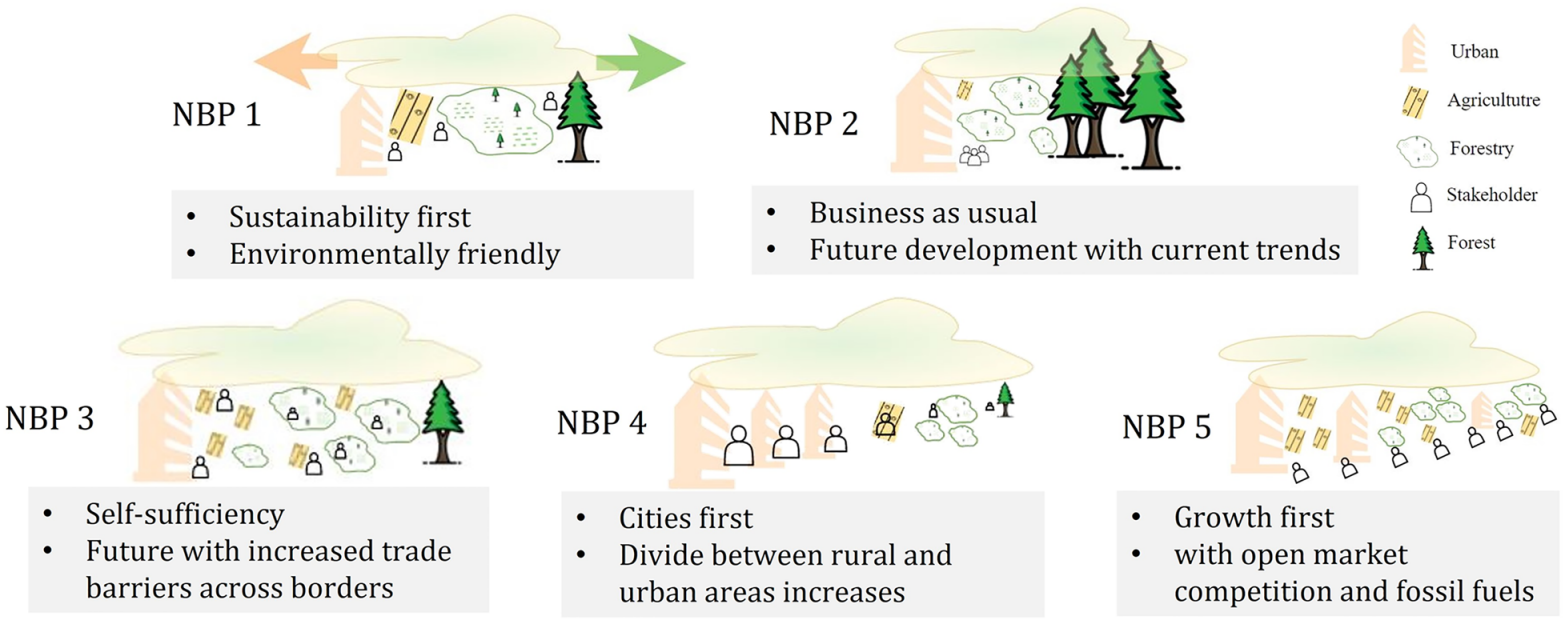
A conceptual diagram of each Nordic Bioeconomy Pathway (NBP) based on the detailed descriptions of NBPs^30^.

This study examined alternative pathways, hereafter known as the Nordic Bioeconomy Pathways (NBPs), to provide a direction to focus on current and future plausible stressors for bioresources. Based on a global level of Shared Socioeconomic Pathways (SSPs)^29^, specific narratives have been developed for the potential expansion of NBPs^30^. The SSPs represent five (SSP 1–5) different storylines for probable future socioeconomic growth to assess the sustainable development context and to focus on the future challenges of mitigation and adaptation^31–34^. Climate policies can also be introduced in SSPs to achieve a consistent radiative forcing level with Representative Concentration Pathways (RCPs)^35^. Concerning NBPs, the current situation is described as the baseline scenario (NBP 0), whereas other NBPs are conceptualised following the main outlines of SSPs^30^. The conceptual framework of each NBP is summarised in Fig. 1.

Narratives of NBPs vary from environment-friendly to open-market competition scenarios. Global socioeconomic drivers of SSPs^32^ have been transformed based on the perspectives of the Nordic region, outlined as land system management (LSM) attributes of NBPs. LSM attributes include (i) Catchment Management Strategy (CMS), and (ii) a combination of Biomass Removal (BMR) and Stand Management (SM) (BMR-SM). Although the qualitative description of LSM attributes explains the socioeconomic conditions in the Nordic regions, quantitative estimates require more case-specific information to understand the consequences of each NBP on water quality. However, not all qualitative LSM attributes are suitable for the quantitative translation of the catchment owing to the catchment topography and the applicability of the catchment modelling tool.

This study aimed to explore the variation in LSM attributes, especially forestry, under different NBPs and how they influence hydrology and water quality in a peatland forestry-dominated Simojoki catchment in northern Finland. The CMS and BMR-SM attributes for each NBP were applied through various processes and scenarios in a calibrated catchment model to understand how the model could reflect hydrological status, nutrients, and SS.

We hypothesized that the individual impacts of LSM attributes would dominate the changes in hydrology and water quality compared with the climate. Specifically, we assumed that the water quality changes would be lower in the sustainable (NBP 1) and business-as-usual (NBP 2) scenarios than in the other NBP scenarios because the quantitative estimates (based on the narratives in Fig. 1) for different LSM attributes were also lower than those in the baseline scenario (NBP 0).

## NBPs and scenarios for Land System Management (LSM) attributes

The scenarios used in this study were quantitative transformations of the NBP storylines for catchment modelling. As shown in Fig. 1, the major goal of each NBP scenario (NBP 1–5) varies from sustainability to the growth of society, covering many socioeconomic drivers^30^. Selecting the specific forestry attributes and translating the quantitative projections of the CMS and BMR-SM attributes were challenging because the interpretation was made from qualitative narratives to numerical values in a particular modelling framework. Thus, this study involved multiple phases in integrating all the processes, including stakeholders, specific tools, and existing catchment models, to project current and future quantifications and effectively set up the pathway.

**Figure 2:**
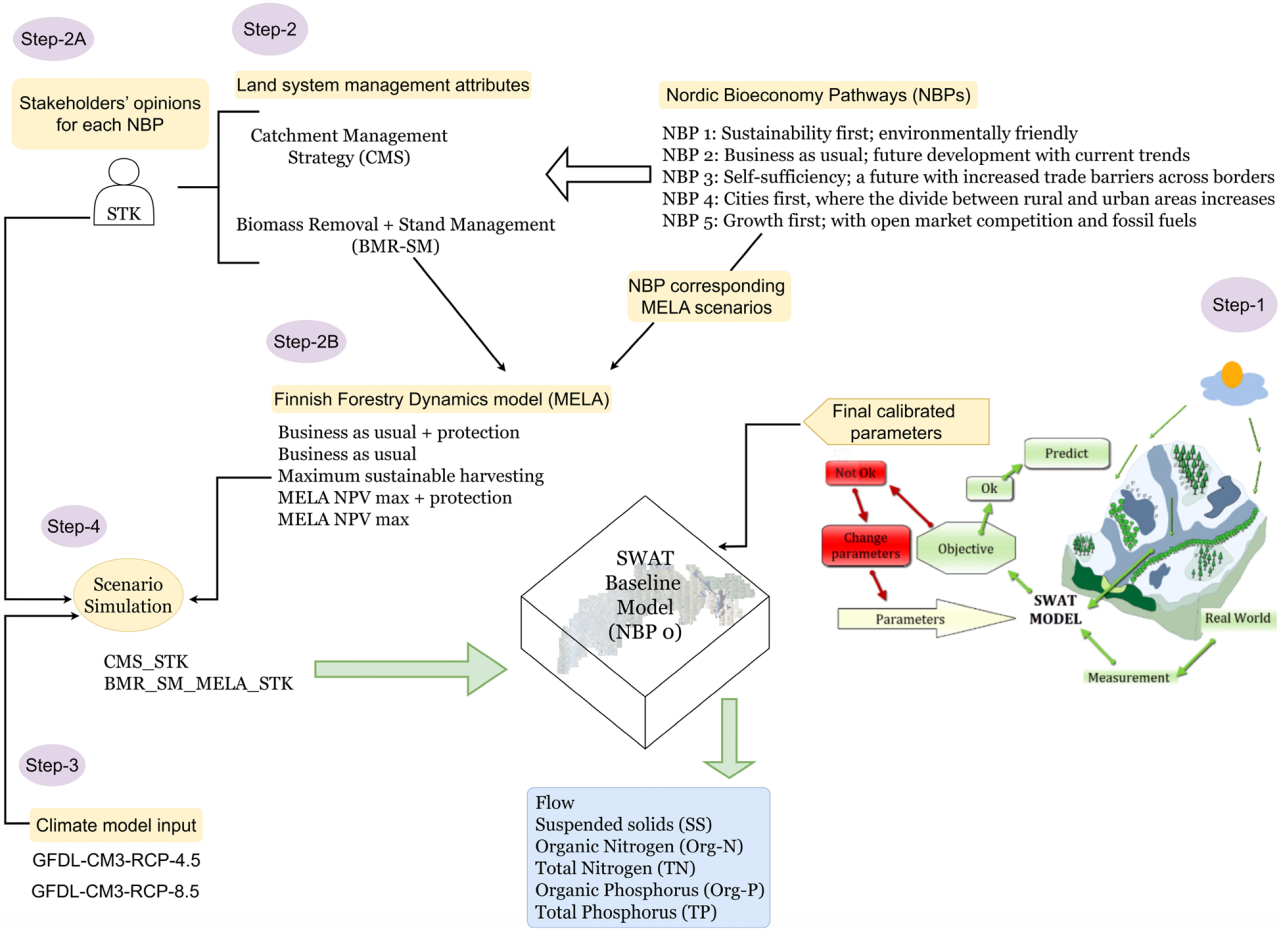
Detailed processes to implement NBP Scenarios.

The stakeholders provided their input for different perspectives to define the probable quantitative projections of the five NBPs on a scale of one to five. Figure 2 represents Step-1 to Step-4 which were followed in this study to implement the NBP scenarios in the Soil and Water Assessment Tool (SWAT) to simulate all scenarios.

### Step-1: catchment and SWAT model (baseline scenario or NBP 0)

The Simojoki river catchment in northern Finland was considered for implementing NBP scenarios as the catchment has intensive peatland forestry activities. Another major reason for selecting this catchment was the availability of the required input to set up the model in SWAT. After simulating the initial model in SWAT, parameters were analysed to calibrate the model in SWAT-Cup. After completing the sensitivity analysis, the parameters were finalised to start the process of NBP scenario implementation. The detailed process and results of the SWAT model are explained in a previous study (Methodology and Results section, forthcoming)^47^.

The calibrated SWAT model of the Simojoki catchment was considered the baseline or reference scenario (NBP 0). The flow, SS, organic nitrogen (Org-N), total nitrogen (TN), organic phosphorus (Org-P), and total phosphorus (TP) of the catchment were modelled for the analysis. Including five years warm-up period, the model was calibrated from 1985 to 2002 and validated from 1998 to 2015. Next, NBP scenarios were implemented over the baseline model and simulated across the selected LSM attributes, and the outcomes were compared with those of the baseline model.

### Step-2: selection of LSM forestry attributes for the Simojoki catchment

Of the six forestry attributes (see SPM-A 1 in Supplementary Materials (SPM)), the following LSM attributes were selected based on the projections of the NBP storylines:

1. Catchment management strategy (CMS)
2. A combination of biomass removal + Stand management (BMR-SM)

CMS and BMR-SM were the most suitable attributes that could be implemented in this study. The other attributes (i.e.; dominant tree species and land cover) were not selected because of the model structure, catchment topography, and the avoidance of conflicts among the attributes. Two different approaches were used to apply each selected LSM forestry attribute to the SWAT baseline model.

- *Step-2A* Involvement of stakeholders (STK) and using their opinions as a percentage of changes in the SWAT catchment model compared with the baseline model.
- *Step 2B* Finnish forest dynamics model MELA to generate output from MELA's corresponding scenarios that resemble NBP scenarios and use the output as an input condition to the SWAT baseline model.

For the CMS attribute, only the percentage of stakeholders, and for the BMR-SM attribute, both stakeholder percentage and MELA's output were used to finalise the input of the scenarios before assigning them to the baseline model.

#### Step-2A: Stakeholder's opinion and quantification of the NBP narratives for SWAT

Two stakeholder groups were invited to participate in the interviews, based on a questionnaire on the attributes of the NBPs. The stakeholders were familiar with different managerial activities in the Simojoki catchment area. Some of them were private forest owners and a maximum of them were actively involved in executing the planning of the forestry actions. A group of forest experts who were leading the bioeconomy transition concept developed the questionnaire, and a sample question regarding the CMS attribute is provided in Online Appendix-A 1 (see Online Appendix). The selected forestry attributes for the five NBPs were explained to the stakeholders. Each attribute was assigned a scale of one to five. They were asked to provide input based on their perception of each NBP (Online Appendix-A 1).

The outcome of each stakeholder group for a specific attribute (i.e., CMS) of each NBP is given in Online Appendix-A 2 with the expert judgment of the expected extremes on the NBP attribute axis used in the interviews. The conversion from the narratives of NBPs to the exact percentage of corresponding attributes depends on a scale of one to five. Based on stakeholders' opinions, expert judgments, and scale, a sample calculation table is provided in Online Appendix-A 3, which represents the detailed calculation of the percentages of certain land uses based on the CMS attribute of each NBP. Online Appendix-A 3 provides an example of a protected land use category. The same process was followed for the calculation of other land use types.

#### Step-2B: Finnish forest dynamics model MELA

MELA is a Finnish forestry dynamics model used to simulate stand development for forest management planning purposes^36^. MELA consists of two parts:

1. An automated stand simulator based on tree-level natural processes and production models and
2. An optimisation package based on linear programming

Publicly available forest inventory data on stand characteristics provided by the Forest Centre were used as input data for MELA simulations.

Stand data did not cover the entire study area. Therefore, the stand-level simulation results were first converted into a grid format (60 m × 60 m). Then the data for the grids that were outside of the original stand data were supplemented with publicly available Multi-Source National Forest Inventory (MSNFI) data which covers all forest areas. Five MELA scenarios were simulated. The results of the MELA scenarios were used to implement NBP scenarios. The links between the NBP and MELA scenarios can be summarized as follows:

- *NBP 1* Sustainability =  > MELA: Business-as-usual + more protection, no ditch network maintenance (DNM) (interest rate, r = 1%)
- *NBP 2* Business as usual =  > MELA: Business-as-usual with ditch network maintenance operations (r = 3%)
- *NBP 3* Self-sufficiency =  > MELA: Maximum sustainable harvesting (r = 3%)
- *NBP 4* Cities first =  > MELA: Net Present Value (NPV) max + protection of forests close to rivers and lakes + protection of other valuable areas (+ restoration) (r = 3%)
- *NBP 5* Growth first =  > MELA: NPV max (r = 3%)

Some limitations were considered before linking the NBPs and MELA scenarios. For NBP 1, the MELA scenario (business-as-usual + more protection) captured some elements of sustainability, including longer rotations due to the low-interest rate level, but not all because continuous cover forestry (CCF) and restoration were unavailable. For NBP 3, it was subjective to say that the maximum sustainable harvesting of MELA represented this scenario. However, this was always a subjective choice regarding the implementation of the scenario for analysis. For NBP 4, the two MELA output scenarios were combined, based on an analysis of the protected grids before using the input into the SWAT. Thus, five different MELA output scenarios were used for different NBPs for the forestry LSM attribute: Biomass removal (BMR) and Stand management (SM).

##### Processing of the MELA output to use in SWAT

The MELA model was simulated using detailed forestry information for each decade from 2031 to 2070 in the Simjoki catchment. For each scenario in MELA, the output was generated as a text file containing unique ID-based rows for multiple MELA variables (approximately 188) in different columns. The coordinates were also provided in columns for each unique ID-based row. Each text file represented a corresponding NBP scenario of 10 years, with a total duration of 40 years. Thus, five text files were representative of the five NBP scenarios. In each text file, there were four columns of the same MELA variable representing 10 years' average value of a specific variable for each decade from 2031 to 2070. Therefore, for each NBP, the first task was to create a spatial entity based on the necessary output variables of MELA for each decade.

Multiple Python scripts (SPM-C) were developed, and several steps were followed to process the MELA output variables (see detailed descriptions in SPM-B and SPM-C). The first task was to form point shapefiles for each unique row from the MELA output scenarios and then clip the corresponding points for each sub-catchment (111) and hydrologic response unit (634 HRUs in the baseline model) of the SWAT model. Next, one central feature (spatial average) of the MELA output scenario was identified to represent each specific HRU as there were multiple points in each HRU. Then, for each decade, all central features were merged (634 rows for 634 HRU) to choose the specific variables from the merged shapefile to assign to the SWAT model's management and plant database file.

#### Finalisation of data processing for selected Land system management (LSM) attribute

##### Catchment management strategy (CMS) input: only from Stakeholders (STK)

Three major land uses were considered while incorporating stakeholder opinions into the SWAT model database. As provided in the last column of Online Appendix-A 3, for each NBP, the total area of each land use type was calculated from the questionnaire. The next task was to calculate the percentage of land use for each NBP scenario. The figure in Online Appendix-A 4 represents the ultimate percentage used to create the land use map for each NBP. The spatial changes were performed mainly near the downstream of the Simojoki catchment to assign the percentage of protected and forestry land use categories.

Five new land use maps were generated, each assigned to the SWAT baseline model to create an individual SWAT model for each NBP. The next task was to simulate the new scenario model in the SWAT-Cup using all the calibrated parameters. Each scenario was simulated from 1985 to 2015 with a 5-year warm-up period, similar to the SWAT baseline model. As shown in Fig. 2, at this stage, the results of the baseline model and all scenarios (NBP) were finalised for the period 1990–2015 for selected nutrients and SS. Regional climate models were also considered for each NBP to determine projections of the NBP scenarios. The integration of climate models and assignment of these models for each NBP are discussed in Step-3.

##### Combined input for Biomass removal (BMR) + Stand management (SM): from both MELA output and stakeholders (STK)

The shapefile for each decade (2031–2070) was merged with the SWAT baseline model database. As MELA had many output variables, not all of them were suitable to directly use in the SWAT model. Thus, the major issue was the selection of MELA output variables that could be used in the SWAT database, and how to use the stakeholder percentage for the BMR and SM attributes. The SWAT baseline model management and plant database files were used to assign changes.

The concept was to use the biomass output from the MELA model and assign these biomass values as the initial biomass in the SWAT management file for each decade and the NBP. Three major output biomass variables were used in the MELA model:

(i) Biomass from the crown (BM_cr_),
(ii) Biomass from the stump and root (BM_sr_), and
(iii) Biomass from the stem (BM_st_) of the tree

The following formula was applied to the SWAT management database to use the output values of each HRU:

1 $$Initial\,biomass=([BMcr] + [BMsr]+ [BMst])*1000/ ([area\_hru])$$

The initially calculated biomass was applied to each HRU after merging the shape file with the SWAT management file. Next, the planting and harvesting operation schedule was used in the management file of the representative (forestry) HRUs. Therefore, stakeholders were asked and based on their views, planting started in May, and for some NBPs, rotation was applied until the end of August.

Stakeholders also provided inputs based on the questionnaire. Compared to the baseline, the amount of BMR and SM attributes varied by 11% for NBP 1, 6.8% for NBP 2, -13.5% for NBP 3, 8.4% for NBP 4, and -16% for NBP 5 (see Online Appendix-A 5 for detailed calculations). When the percentage decreased, there were two NBPs (NBP 3 and 5). Therefore, to implement the percentage variation of BMR and SM in the SWAT management database, the harvesting of the plants and rotation continued for each decade, as MELA output was representative only for a decade. The average rotation of tree growth and planting was also considered because the SM was almost identical to the BMR. For the other NBPs, the percentage increment was adjusted with the initial biomass and rotation of planting only in the forest HRUs.

After setting up the management and plant database files for each decade, an individual SWAT scenario model was finalised for each NBP, and the new scenario model was simulated in SWAT-Cup with all calibrated parameters. Each scenario model was simulated for each decade from 2031 to 2070 with a 10-year warm-up period because the MELA model output was representative for each decade. Regional climate models were integrated for each decade for each NBP scenario. As the MELA model output was available from 2031 to 2070, it was not feasible to simulate the model from 1990 to 2015. Therefore, regional climate models were integrated with the baseline model to compare the future projections of the baseline model and NBP scenario of each decade (see Step-3 for a detailed explanation of the regional climate models).

### Step-3: integration of regional climate models

For the Simojoki catchment, representative concentration pathways (RCPs) from different regional climate models are available from the Finnish Meteorological Institute (FMI). RCPs are greenhouse gas concentration trajectories to describe different climate futures, depending on the volume of greenhouse gases (GHG) emitted in the years to come. The RCPs are labelled as RCP-2.6, RCP-4.5, RCP-6, and RCP-8.5 which define a possible range of radiative forcing values in the year 2100 (2.6, 4.5, 6, and 8.5 W/m^2^ respectively)^37^.

Naturally and based on the narratives of NBPS (Fig. 1), not every combination of NBPs and RCPs is feasible. For example, NBP 3 with a radiative forcing of 1.9 and 2.6 W/m^2^ was not feasible in integrated assessment models due to regional rivalry obstructing global coordination of mitigation efforts^31^. Thus, based on the experts' opinions and experiences from previous projects and as the main interest of this study was to explore the consequences of medium and extreme emission scenarios, RCP-4.5 and RCP-8.5 were selected. The Geophysical Fluid Dynamics Laboratory (GFDL) model was also found most suitable in the study area for the mentioned RCPs.

A sample comparison was made between available model data and historical records from 1980 to 2015 (baseline model simulation period 1985–2015). Monthly and yearly comparison plots of the sample weather data were provided in the SPM-D to portray how different meteorological data (i.e., rainfall (P), relative humidity (RH), and maximum and minimum temperatures (T_max_ and T_min_) varied with the timeline.

After collecting the climate data of different RCPs from FMI, the data were processed to provide input into the SWAT-Cup to implement the RCPs in each NBP scenario, with the final calibrated parameters.

### Step-4: scenario simulation

For each LSM attribute, the developed scenarios were simulated based on the final input from stakeholders, the MELA model, or both. Therefore, for the Catchment management strategy (CMS), there were five scenarios (NBP 1–5) for NBPs from 1990 to 2015. When data from RCPs were integrated with each NBP, there were 10 more scenarios for the CMS attribute (see Online Appendix-A 6).

For the combination of Biomass Removal and Stand Management (BMR-SM), each NBP scenario was simulated for each decade. Thus, there were 40 scenarios for all NBPs (NBP 1–5), including RCP-4.5 and RCP-8.5.

The results of all scenarios were analysed and compared with the baseline model for flow, SS, Org-N, TN, Org-P, and TP (Detailed python scripts are provided in SPM-E).

Later, for each modelled response variable, the statistical significance of the outcomes was tested between baseline (NBP 0) and each NBP scenario separately using the non-parametric Mann-Whitney U-test with a 0.05 significance level. The statistical analysis assumed that the median of any modelled response variable of NBP 0 was equal to the median of the same variable of any NBP from NBP 1 to NBP 5.

## Results

**Figure 3:**
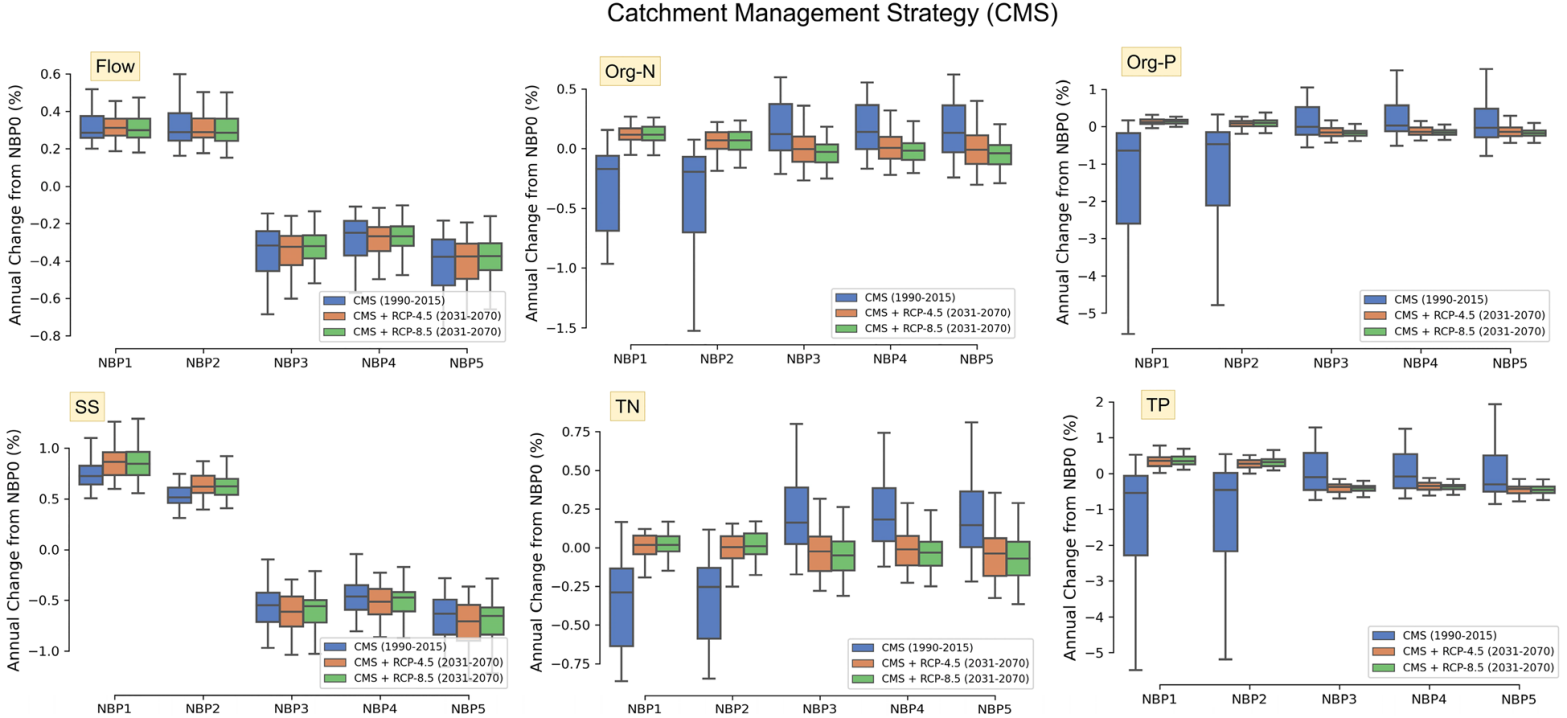
Changes in annual flow, nutrients, and SS for the CMS attribute of each NBP compared to NBP 0.

**Figure 4:**
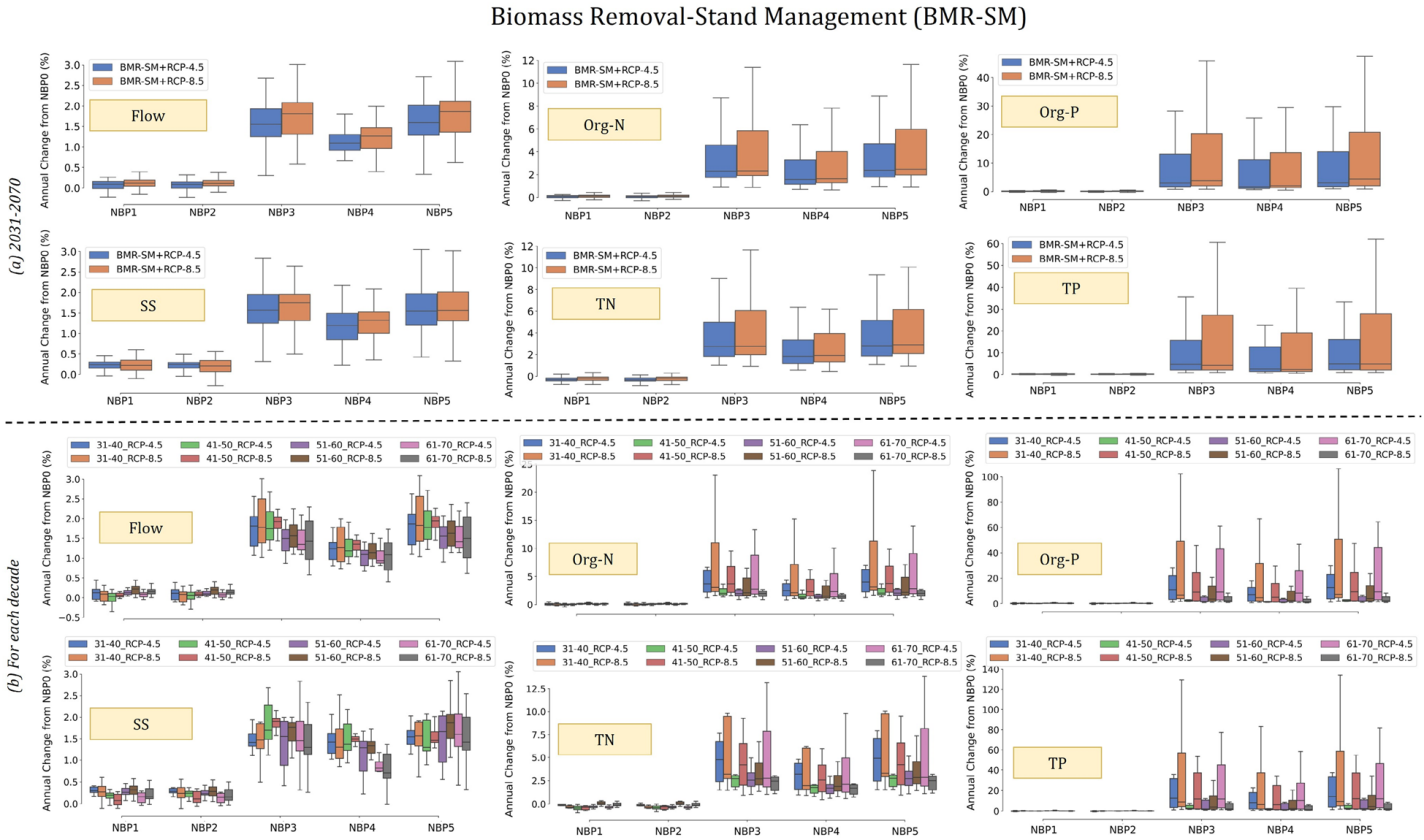
Variations in annual flow, nutrients, and SS for the BMR-SM attribute of each NBP compared to NBP 0. The top panel represents the variations of the modelled response variables from 2031 to 2070 whereas the bottom panel is showing the results of each decade.

Tables 1 and 2 summarise how each NBP scenario varied from the baseline scenario (NBP 0) (see Online Appendix-A 6 for all scenarios). The calibrated SWAT model for the Simojoki catchment was the baseline scenario. Two GHG emission pathways (RCP-4.5 and RCP-8.5) were selected to understand the impacts of climate and each attribute for different periods (Tables 1 and 2 and Figs. 3 and 4). The results highlight the variations in flow, SS, organic nitrogen (Org-N), total nitrogen (TN), organic phosphorus (Org-P), and total phosphorus (TP) in the catchment across different scenarios.

**Table 1:** Summary results of each NBP compared to NBP 0, for Catchment management strategy attribute from Stakeholders (CMS-STK).

Flow, nutrients and SS	NBP 1			NBP 2			NBP 3			NBP 4			NBP 5		
	Sustain- ability first			Conventional first			Self-sufficiency first			City first			Growth first		
	C	R1	R2	C	R1	R2	C	R1	R2	C	R1	R2	C	R1	R2
Flow	+	+	+	+	+	+	−	−	−	−	−	−	−	−	−
SS	+	+	+	+	+	+	−	−	−	−	−	−	−	−	−
TN	−	−	+	−	−	+	+	0	−	+	0	−	+	0	−
TP	−	+	+	−	+	+	+	−	−	+	−	−	+	−	−
Org-N	−	+	+	−	+	+	+	0	−	+	0	−	+	0	−
Org-P	−	0	+	−	0	+	+	−	−	+	−	−	+	−	−

C represents the period from 1990 to 2015, as same as the baseline scenario’s period, R1 represents attribute + RCP–4.5 from 2031 to 2070, and R2 shows attribute + RCP–8.5 from 2031 to 2070. The symbol ‘0’ represents no change, ‘ + ’ represents an increase of the scenario up to 10%, and ‘-’ represents a decrease up to 10%.

**Table 2:** Summary results of each NBP compared to NBP 0, for Biomass removal and Stand management attribute from MELA and Stakeholders (BMR-SM-MELA-STK).

Flow, nutrients and SS	NBP 1		NBP 2		NBP 3		NBP 4		NBP 5	
	R1	R2	R1	R2	R1	R2	R1	R2	R1	R2
Flow	+	+	+	+	+	+	+	+	+	+
SS	+	+	+	+	+	+	+	+	+	+
TN	+	+	+	+	+	+	+	+	+	+ +
TP	+	+ +	+ +	+ +	+ +	+ +	+ +	+ +	+ +	+ +
Org-N	+	+	+	+	+	+	+	+	+	+
Org-P	+	+ +	+	+ +	+ +	+ +	+	+ +	+ +	+ +

R1 represents attribute + RCP–4.5, and R2 shows attribute + RCP–8.5 for the entire period from 2031 to 2070, not for each decade as it was simulated. The symbol ‘ + ’ represents an increase of the scenario up to 10%, and ‘ +  + ’ represents an increase of that scenario greater than 10%.

On average, the change in the annual values of each NBP was compared with the annual values of NBP 0. Four different symbols were selected to categorise the variation in each scenario, and are summarized as follows:

(i) 0 represents no change
(ii)  + represents an increase <  = 10%.
(iii) – represents a decrease <  = 10%
(iv)  +  + represents an increase > 10%

Owing to differences in the data sources in the baseline model and uncertainties in the climate input, the results of the scenarios are presented as percentage changes over the baseline scenario as opposed to absolute values. To specify how CMS and BMR-SM affected flow, nutrients, and SS in each NBP, variations are provided in Figs. 3 and 4, along with the results of regional climate models.

Although the increase and decrease of each modelled response variable were small compared to NBP 0, the outcomes of each scenario showed statistically significant differences for each of the modelled response variables from the Mann-Whitney U test result (see Online Appendix-A 7). For example, for TN, between the results of NBP 0 and any NBP analysed in this study, the P-value was less than 0.05 which indicated that the median of TN in NBP 0 was not equal to the median of TN in another NBP scenario. This situation was the same for other modelled response variables for both CMS and BMR-SM attributes.

### Catchment management strategy from stakeholders (CMS-STK)

The annual flow in Simojoki increased by 0.3% for NBP 1 and NBP 2. Based on stakeholder opinions, as there were fewer forestry actions in NBP 1 and NBP 2, the evapotranspiration of the entire catchment decreased, thus increasing streamflow. Even with climate input, the increases in annual flow in scenarios NBP 1 and NBP 2 were almost identical.

On average, a flow reduction was expected for the NBP 3, NBP 4 and NBP 5 scenarios because of greater self-sufficiency and economic growth-related activities. The land uses of these NBPs differed by 5–10% with NBP 0. Thus, the flow decreased by 0.25–0.55%. Although for different NBPs, the patterns of SS were almost the same as the changes in flow, the differences in percentage were higher compared to the NBP 0.

The annual changes in Org-N and TN were almost the same compared to NBP 0 for the CMS attribute. From 1990 to 2015, TN decreased by approximately 0.3% for NBP 1 and NBP 2, whereas an increase of approximately 0.15% was observed for the other NBPs. Owing to the climate input, the changes in TN in all NBP scenarios were close to 0% from 2031 to 2070.

The decrease in forestry and increased protected coverage resulted in a 0.5% decrease in Org-P and TP for NBP 1 and NBP 2. However, both nutrients increased slightly during 2031–2070 when the climate input was integrated with this attribute.

### Biomass removal and stand management, both from MELA output and stakeholders (BMR-SM-MELA-STK)

As there was a substantial change between the biomass and stand-level inputs of BMR-SM in each NBP, the changes in annual flow were also substantial compared to NBP 0. On average, for the entire period, the flow increased by 1.4% (Fig. 4, top panel). Based on stakeholders' opinions, there were always biomass removal operations in forestry hydrologic response units (HRUs) in SWAT. Thus, the flow increased from slight to mild (0.1–3%) for all the NBP scenarios (see the example questionnaire in Online Appendix-A 1). The increases in NBP 3 and NBP 5 were greater (> 1.5%) than those in other NBP scenarios. Because the main goal of both scenarios was to focus on growth, a higher flow generated for these NBP scenarios was expected. For sustainable scenarios (i.e., NBP 1), the annual flow changes were almost identical to those of NBP 0.

The SS variations were similar to those observed in the flow. However, the NBP 5 scenario resulted in the highest increase (3%), whereas the NBP 1 scenario showed the lowest change (~ 0.2%) compared to the NBP 0.

For RCP-4.5, both Org-N and TN increased by approximately 8.5% for NBP 3 and the other NBPs followed this trend. For RCP-8.5, a 10% increase in NBP 5 was observed. The initial biomass input into the SWAT varied for each decade from 2031 to 2070. Thus, the overall change in TN varied from 2 to 11% for all NBP scenarios except for NBP 1 and NBP 2.

Higher biomass removal and stand management resulted in the highest increase in TP. The change was visible for NBP 3 to NBP 5, whereas for the other NBPs, the change was almost 0% compared to NBP 0 for both regional climate models.

### Interpretation of NBP scenarios

Based on the concepts of NBPs (see Fig. 1), SWAT catchment modelling of NBP scenarios can be interpreted as follows:

- Shifting towards a more sustainable society in NBP 1 led to a focus on fewer forestry activities, resulting in a decrease in TN and TP. However, for the flow and SS, the situation was the opposite. When biomass was removed, the variability in TN and TP was higher for BMR-SM and RCP-8.5, than for the combined input of biomass and RCP-4.5.
- Currently, approximately 57% of the drained peatland area is in the Simojoki catchment. Under these conditions, the scenario results for NBP 2 showed a pattern similar to that of NBP 1. The current biomass removal rate and aged stands resulted in a slightly higher climate effect than sustainable NBP 1.
- Forest management will be intensive as biomass grows to a certain extent in NBP 3. Thus, forest harvesting resulted in a high increase in TN and TP. Even with climate input, it was clear that changes in nutrients and SS would result in significant challenges for mitigation and adaptation.
- When the gap increased between the urban and rural sites in NBP 4, TN increased, followed by TP. However, for the BMR-SM attribute, the biomass removal percentage varied between the input of the stakeholders (6.8%) and the output of the MELA model (8.4%).
- High economic growth and rapid technological development in NBP 5 resulted in the highest increases in nutrients and SS. As the pathways are more material-intensive, climatic challenges would be higher.

In summary, the land system management attributes for different bioeconomy pathways dominated the changes in hydrology and water quality compared to the climate.

## Discussion

Alternative pathways for bioresource use have the potential to lead to plausible stressors in the future in Nordic catchments. The input differences in multiple land system management attributes affected the hydrology and water quality of the catchment more than the climate. The LSM attributes resulted in slight to mild fluctuations in the hydrology and water quality of the catchment owing to the conceptual differences in the NBPs. The assessment process of comparing different NBP scenario results with NBP 0 was used to reproduce trends for the current period and the next 40 years, without systematic deviation from the measurements. For the BMR-SM attribute, the model was simulated with each attribute and climate input, and the results retrieved for the CMS attribute clearly showed that the distinct impact of the climate model was negligible. For both climate models, different RCP scenarios had very little effect on any modelled response variable.

The export of nutrients and SS for different NBP scenarios form an extensive range around LSM forestry attributes owing to the bottom-up approach of quantification from stakeholders or the MELA model. However, due to past drainage activities in the Simojoki catchment, changes in the total organic carbon (strong correlation with nitrogen) were primarily driven by climate rather than forestry^38^. Owing to the combined changes in land use and climate, no particular effects were found on phosphorus exports in smaller catchments^39^. Our study indicates that political decisions from different land use practices at the catchment level can impact hydrology and water quality, and the results from Simojoki can be used to guide more sustainable forestry actions at the catchment level.

Reduced stand management and biomass removal led to lower flow and export of nutrients and SS in the sustainable (NBP 1) and business-as-usual (NBP 2) scenarios, which supported the hypothesis of this study. For the other NBP scenarios, the flow and export of nutrients and SS increased with decreased evapotranspiration. Owing to slow tree growth and long forest rotations, it is sometimes difficult to determine the reason for changes in nutrients and SS in large catchments^40^. For example, in NBP 4, when biomass and climate data were integrated into the SWAT model, a large portion of nitrogen was removed from the harvested products, and a certain amount of phosphorus was retained in the soils. Although the current forest management operation for land use, existing biomass, and removal from the system were validated against the baseline model (Results section, forthcoming)^44^, the increase in nutrients and SS export was still noticeable, which was unexpected.

Of the five pools in the SWAT^41^, TN and TP exchanges mostly varied in the organic fresh pool related to the initial biomass in the forestry HRUs and the removal of biomass after harvesting also increased nutrient transport^42^. Because NBP 3 and 5 had a higher ratio of biomass removal in the catchment, the export of nutrients and SS was also higher in those NBPs. A similar analysis was also applicable for organic and inorganic nutrients, where organic nutrients were the major drivers throughout the catchment, which varied in the same way as total nutrients^43^.

Although this study provides the individual effects of varying LSM attributes, one of its limitations is the determination of the joint impact of all attributes on different NBPs if they are applied together to the catchment model. As the input data were taken from multiple sources (stakeholders, MELA), and the period of the input data differed greatly, it was only possible to simulate the model for specific LSM attributes. However, to know how the scenario would look if all attributes were integrated into the SWAT model at a time, Tables 1 and 2 provide a conceptual understanding of the response variables for the transition towards NBPs. There can be concern about the significance of the results, especially regarding the large error bars for phosphorus and using the same symbol (‘ + ’) in Tables 1 and 2 to categorise the increase of the variable up to 10%. The increase in phosphorus was more evident in the BMR-SM attribute. As mentioned in the SWAT baseline model study (forthcoming)^44^, peat soils contain more than 50% phosphorus, and runoff in drained peat soils was higher than that in undisturbed soils. The spatial biomass output retrieved from the MELA model and the management operations applied in SWAT may have affected the Org-P and TP outputs in the scenarios. We acknowledge that the annual changes in phosphorus did not match our expectations. However, based on the output of all modelled response variables and scenarios, maybe the most sustainable bioeconomy pathways may not be the most probable option to implement in the Simojoki catchment.

### Future perspectives

To achieve good ecological status for all surface water bodies, the requirements for the next planning cycle of the Water Framework Directive (WFD 2022–2027)^45^ will be followed in Finland. In the Simojoki catchment, 11.3% of the nitrogen and 1.7% of the phosphorus loads need to be reduced by 2027. In this study, in maximum cases, the projections of nutrients in each NBP were higher than the estimated target^45^. However, for NBP 1 and NBP 2, the decreases in TN and TP were closer to the targets that need to be achieved by 2027. It should be noted that the scenario results presented in this study showed variations based on the changes in specific attributes.

Many processes at the catchment level are already active in achieving the WFD targets. Concerns have been raised about whether managerial actions are too slow to reach goal^46^. Demand is also increasing to provide a plausible solution for balancing managerial actions and WFD goals^47^. The approach presented in this study can benefit from this perspective by identifying sensitive zones in a catchment with peatland forestry, and where to implement managerial actions.

**Figure 5:**
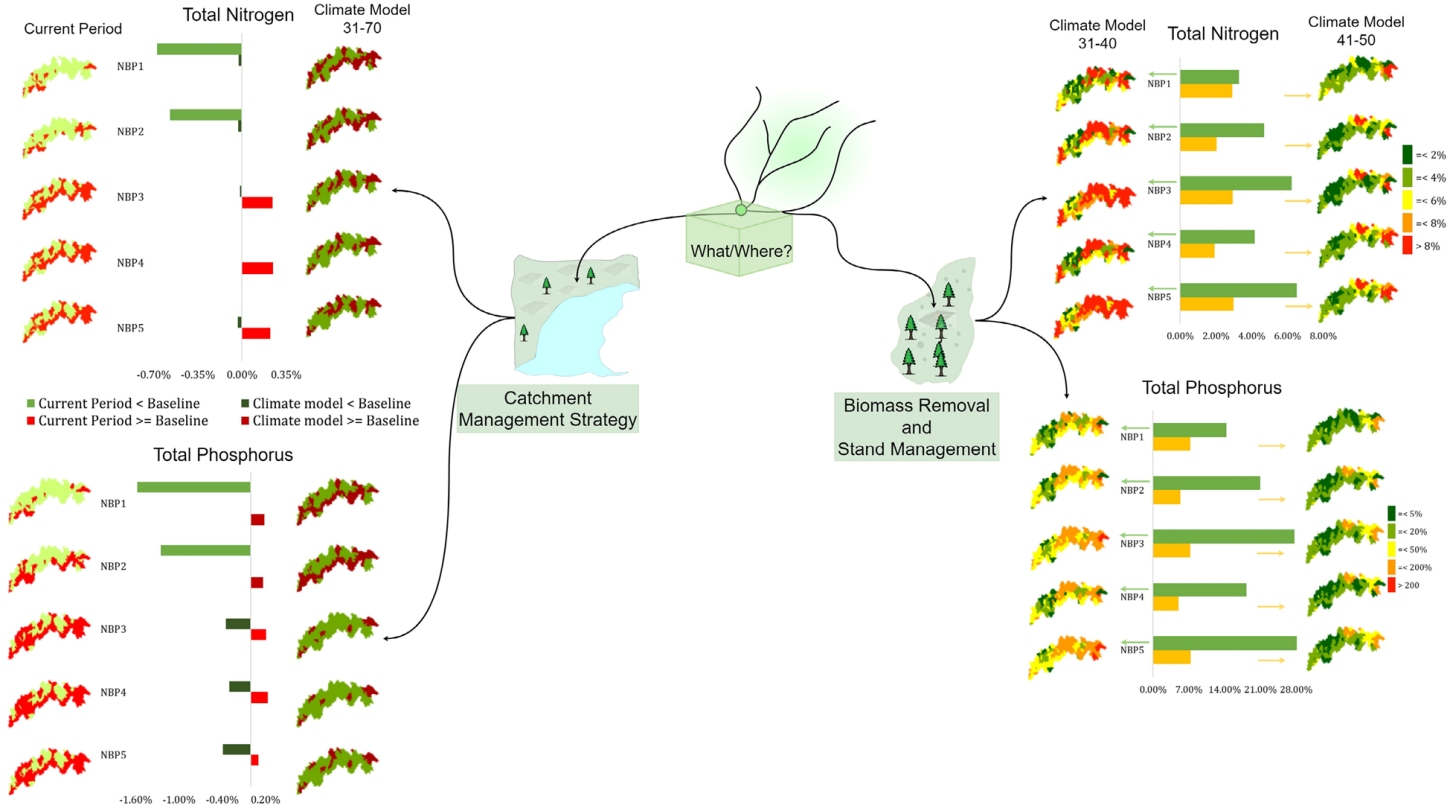
A recommended chart to follow for taking a managerial decision.

Figure 5 shows the overall choices and directions for selecting a specific forestry attribute. For instance, in the left-hand figure, the spatial variations in total nitrogen and total phosphorus for the current period and from 2031 to 2070 after providing the catchment management strategy and climate input created an option to identify the sensitive areas within the catchment. The authority can focus on which location in the catchment the loading will be reduced and where extreme loading can occur, compared to the baseline scenario. If the managerial authority is interested in understanding the impact of biomass removal and stand management, the right-hand figure can be used. For example, an authority can focus on this attribute if there is a demand to maintain a certain threshold for aquatic species at any location within the catchment. They can check which location in the catchment they can choose to reduce nitrogen and phosphorus loads in the upcoming decade from 2031 to 2040 and 2041–2050. Thus, the corresponding authority can take rapid action.

## Conclusions

Implementing the bioeconomy must not happen at the cost of the environment. This study aimed to explore what could be done or what needed to be known and whether we should proceed in our current direction or take an alternative pathway. It will depend on the trajectory whether the distribution of land use and biomass will be proportional or change greatly based on the focus of the society.

In the sustainability (NBP 1) scenario, shifts in land use type were often the most pronounced. In contrast, a societal trajectory with limited environmental awareness (NBP 3 or NBP 5) would likely have impacts on hydrology and water quality. Thus, in these scenarios, the targeted green goals would be difficult to achieve. However, considering the current context of the political and economic situation, forestry activities and the use of bioresources are highly relevant to regional planning. Although the study was investigated at a local scale, it is likely to apply our approach to forests and similar land uses. Thus, the changes in forestry areas and biomass removal due to the opinions of stakeholders and the MELA model reflected the following changes:

- Implementing bioeconomy pathways emphasising sustainability (NBP 1) would likely decrease the export of nutrients and SS.
- The business-as-usual (NBP 2) scenario also followed the same trend as sustainability, with the decreased flow and increased evapotranspiration.
- In contrast, bioeconomy development drawing on the increased use of renewable resources owing to the economic growth or self-sufficiency aim (NBP 3 or NBP 5) would probably have increased the nutrients and exports of SS.
- Accounting for climate, the analysis clearly showed that the distinct impact of climate had very little effect on any modelled response variables.

However, forestry activities depend on the scenario and local interpretation of the area. Thus, from the results of the scenarios in the Simojoki catchment, it can be said that the most sustainable NBPs may not be the most probable option for implementing bioeconomy pathways.

Overall, the approach to understanding the bioeconomy impacts of foreseen land use management is beneficial, especially in identifying the spatial zones of excessive loading in a peatland forestry-dominated catchment compared to the current conditions.

## Supplementary Information

Supplementary Information 1.

Supplementary Information 2.

## Data Availability

The data that support the findings of this study are available upon reasonable request from the authors (contact: joy.bhattacharjee@oulu.fi). Codes and other relevant information are available in supplementary materials.
