# Supplementary material for: Impacts on water quality in the peatland dominated catchment due to foreseen changes in Nordic Bioeconomy Pathways
Source: Sci Rep. 2023 Apr 18;13:6283. doi: 10.1038/s41598-023-33378-7 (PMC10113390; doi:10.1038/s41598-023-33378-7)
Supplement: Supplementary file 1 — Supplementary Information 1. [file 41598_2023_33378_MOESM1_ESM.docx]

# Appendix-A

Impacts on water quality in the peatland dominated catchment due to foreseen changes in Nordic Bioeconomy Pathways

Joy Bhattacharjee^1*^, Hannu Marttila^1^, Eugenio Molina Navarro^2^, Artti Juutinen^3^, Anne Tolvanen^3^, Arto Haara^4^, Jouni Karhu^3^ and Bjørn Kløve^1^

^1^Water, Energy and Environmental Engineering Research Unit, PO Box 4300, 90014 University of Oulu, Oulu, Finland.

^2^Geology, Geography and Environment Department, University of Alcalá. Ctra. Madrid-Barcelona Km. 33.6, 28805, Alcalá de Henares, Madrid, Spain.

^3^Natural Resources Institute Finland (LUKE), Oulu, Finland.

^4^Natural Resources Institute Finland (LUKE), Joensuu, Finland.

*Corresponding author at Water, Energy and Environmental Engineering Research Unit, PO Box 4300, 90014 University of Oulu, Oulu, Finland. E-mail: [joy.bhattacharjee@oulu.fi](mailto:joy.bhattacharjee@oulu.fi)


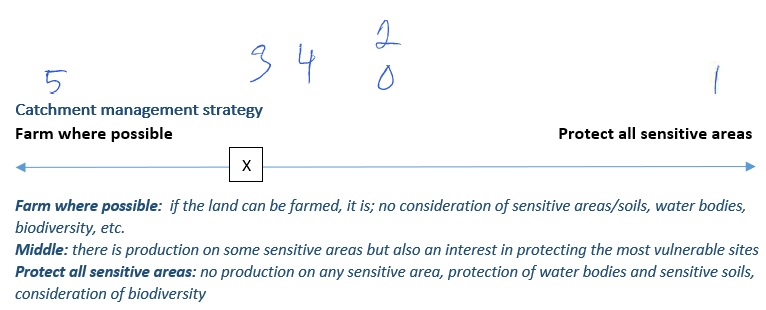


Appendix-A 1. Example of CMS attribute for which stakeholders provided input based on the projection of the NBP scenario

Appendix-A 2. Example of an expert’s evaluation of the ranges of percentages for different landuse categories for CMS attribute

| Landuses | Min | Current | Max |
| --- | --- | --- | --- |
| Agriculture | 0.5% | 3% | 10% |
| Forestry | 65% | 75% | 80% |
| Protection | 5% | 14% | 30% |

Appendix-A 3. An example of the process used to generate the percentage of LU for each NBP for the CMS attribute in the Simojoki catchment.

| Applying min, present, and max percentages for Protected landuse | | | | | | | | | | | |
| --- | --- | --- | --- | --- | --- | --- | --- | --- | --- | --- | --- |
| Protected landuse | | | | | Area-based on the percentage provided in Appendix-A 2 | | | | | | |
| Baseline constant value (expert evaluation) | | | | | 442.4 | | | | | | |
| Expert evaluation of the minimum value | | | | | 158 | | | | | | |
| Expert evaluation of the maximum value | | | | | 948 | | | | | | |
| For attribute #4 | Group 3 | Group 4 | Fin-  land | Index  0-100 | Scaled only for increasing changes (right) | Index 100-0 | Scaled only for decreasing changes (left) | Merged increasing and decreasing values | % Change from baseline | Change in percentage from baseline | New  Area |
| NBP 0 | -1 | 1 | 0 | 50 | 2370 | 50 | 2370 | 2370 | 100.0 | 0.0 | 2370 |
| NBP 1 | 3 | 4 | 3.5 | 85 | 2504.3 | 15 | 2322.6 | 2504.3 | 105.7 | 5.7 | 2504.3 |
| NBP 2 | -1 | 2 | 0.5 | 55 | 2456.9 | 45 | 2227.8 | 2456.9 | 103.7 | 3.7 | 2456.9 |
| NBP 3 | -2 | -2 | -2 | 30 | 2417.4 | 70 | 2148.8 | 2148.8 | 90.7 | -9.3 | 2148.8 |
| NBP 4 | -1 | -0.5 | -0.75 | 42.5 | 2437.15 | 57.5 | 2188.3 | 2188.3 | 92.3 | -7.7 | 2188.3 |
| NBP 5 | -4 | -3 | -3.5 | 15 | 2393.7 | 85 | 2101.4 | 2101.4 | 88.7 | -11.3 | 2101.4 |


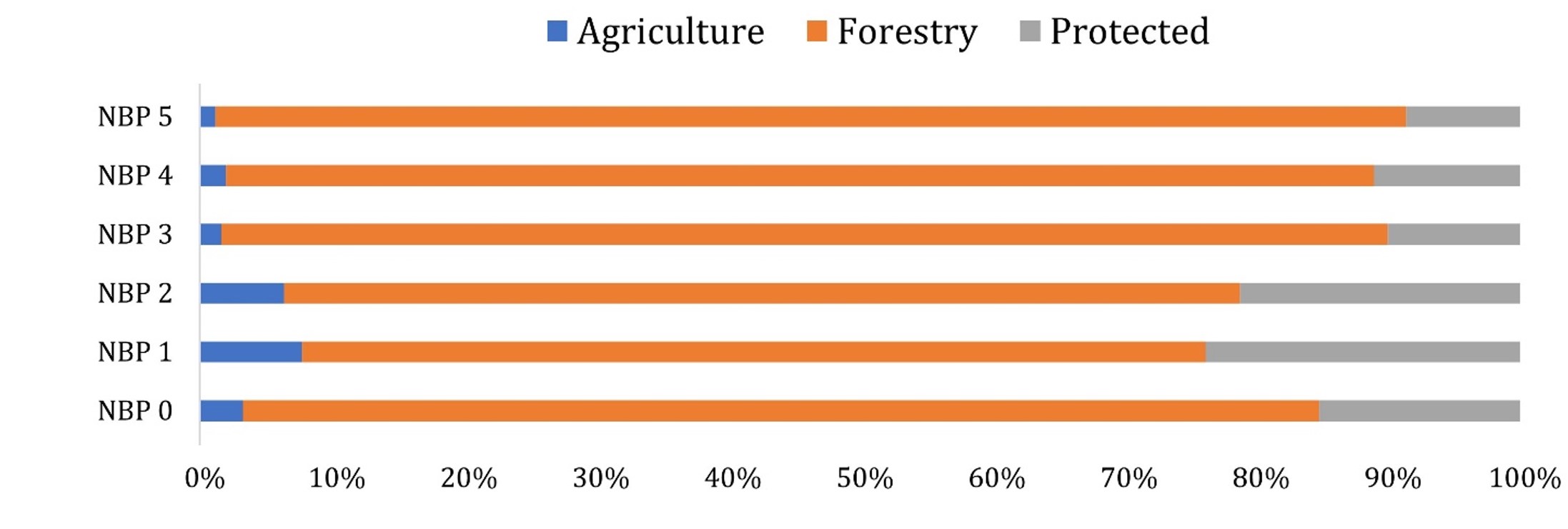


Appendix-A 4. Percentages of considered landuses that were implemented as landuse maps of each NBP in SWAT.

Appendix-A 5. Combination of stakeholder opinions about biomass removal and stand management options

| For  attribute#3 | Group  3 | Group  4 | Finland | Index  0-100 | Scaled only for  increasing changes (right) | Index  100-0 | Scaled only  for  decreasing changes  (left) | Merged increasing  and decreasing values | % change  from baseline | Change in  percentage from baseline |
| --- | --- | --- | --- | --- | --- | --- | --- | --- | --- | --- |
| Baseline | -2 | 0 | -1 | 40 | 62 | 10 | 62 | 62 | 100.0 | 0.0 |
| NBP 1 | 3 | 4 | 3.5 | 85 | 68.8 | -35 | 60.2 | 68.8 | 111.0 | 11.0 |
| NBP 2 | -1 | 1.5 | 0.25 | 52.5 | 66.2 | -2.5 | 56.3 | 66.2 | 106.8 | 6.8 |
| NBP 3 | -3 | -1 | -2 | 30 | 64.4 | 20 | 53.6 | 53.6 | 86.5 | -13.5 |
| NBP 4 | 2 | 1 | 1.5 | 65 | 67.2 | -15 | 57.8 | 67.2 | 108.4 | 8.4 |
| NBP 5 | -5 | -1.5 | -3.25 | 17.5 | 63.4 | 32.5 | 52.1 | 52.1 | 84.0 | -16.0 |

Appendix-A 6. Total number of scenarios considered in this study to analyse flow, TN, TP, SS, Org-N, and Org-P

| Land system management attribute (LSM) | NBPs | Model | Period | Scenario Name |
| --- | --- | --- | --- | --- |
| Catchment Management Strategy (CMS): only from Stakeholders (STK)  (CMS-STK) | NBP 1 | Baseline | 1990-2015 | CMS- STK- NBP 1 |
|  |  | GFDL RCP-4.5 | 2031-2070 | CMS- STK- NBP 1- RCP-4.5 |
|  |  | GFDL RCP-8.5 | 2031-2070 | CMS- STK- NBP 1- RCP-8.5 |
|  | NBP 2 | Baseline | 1990-2015 | CMS- STK- NBP 2 |
|  |  | GFDL RCP-4.5 | 2031-2070 | CMS- STK- NBP 2- RCP-4.5 |
|  |  | GFDL RCP-8.5 | 2031-2070 | CMS- STK- NBP 2- RCP-8.5 |
|  | NBP 3 | Baseline | 1990-2015 | CMS- STK- NBP 3 |
|  |  | GFDL RCP-4.5 | 2031-2070 | CMS- STK- NBP 3- RCP-4.5 |
|  |  | GFDL RCP-8.5 | 2031-2070 | CMS- STK- NBP 3- RCP-8.5 |
|  | NBP 4 | Baseline | 1990-2015 | CMS- STK- NBP 4 |
|  |  | GFDL RCP-4.5 | 2031-2070 | CMS- STK- NBP 4- RCP-4.5 |
|  |  | GFDL RCP-8.5 | 2031-2070 | CMS- STK- NBP 4- RCP-8.5 |
|  | NBP 5 | Baseline | 1990-2015 | CMS- STK- NBP 5 |
|  |  | GFDL RCP-4.5 | 2031-2070 | CMS- STK- NBP 5- RCP-4.5 |
|  |  | GFDL RCP-8.5 | 2031-2070 | CMS- STK- NBP 5- RCP-8.5 |
| A combination of Biomass removal + Stand management (BMR-SM)  (both from MELA output and Stakeholders)  (BMR-SM-MELA-STK) | NBP 1 | GFDL RCP-4.5 | 2031-2040 | BMR-SM-MELA-STK-NBP 1- RCP-4.5-2031-40 |
|  |  |  | 2041-2050 | BMR-SM-MELA-STK-NBP 1- RCP-4.5-2041-50 |
|  |  |  | 2051-2060 | BMR-SM-MELA-STK-NBP 1- RCP-4.5-2051-60 |
|  |  |  | 2061-2070 | BMR-SM-MELA-STK-NBP 1- RCP-4.5-2061-70 |
|  |  | GFDL RCP-8.5 | 2031-2040 | BMR-SM-MELA-STK-NBP 1- RCP-8.5-2031-40 |
|  |  |  | 2041-2050 | BMR-SM-MELA-STK-NBP 1- RCP-8.5-2041-50 |
|  |  |  | 2051-2060 | BMR-SM-MELA-STK-NBP 1- RCP-8.5-2051-60 |
|  |  |  | 2061-2070 | BMR-SM-MELA-STK-NBP 1- RCP-8.5-2061-70 |
|  | NBP 2 | GFDL RCP-4.5 | 2031-2040 | BMR-SM-MELA-STK-NBP 2- RCP-4.5-2031-40 |
|  |  |  | 2041-2050 | BMR-SM-MELA-STK-NBP 2- RCP-4.5-2041-50 |
|  |  |  | 2051-2060 | BMR-SM-MELA-STK-NBP 2- RCP-4.5-2051-60 |
|  |  |  | 2061-2070 | BMR-SM-MELA-STK-NBP 2- RCP-4.5-2061-70 |
|  |  | GFDL RCP-8.5 | 2031-2040 | BMR-SM-MELA-STK-NBP 2- RCP-8.5-2031-40 |
|  |  |  | 2041-2050 | BMR-SM-MELA-STK-NBP 2- RCP-8.5-2041-50 |
|  |  |  | 2051-2060 | BMR-SM-MELA-STK-NBP 2- RCP-8.5-2051-60 |
|  |  |  | 2061-2070 | BMR-SM-MELA-STK-NBP 2- RCP-8.5-2061-70 |
|  | NBP 3 | GFDL RCP-4.5 | 2031-2040 | BMR-SM-MELA-STK-NBP 3- RCP-4.5-2031-40 |
|  |  |  | 2041-2050 | BMR-SM-MELA-STK-NBP 3- RCP-4.5-2041-50 |
|  |  |  | 2051-2060 | BMR-SM-MELA-STK-NBP 3- RCP-4.5-2051-60 |
|  |  |  | 2061-2070 | BMR-SM-MELA-STK-NBP 3- RCP-4.5-2061-70 |
|  |  | GFDL RCP-8.5 | 2031-2040 | BMR-SM-MELA-STK-NBP 3- RCP-8.5-2031-40 |
|  |  |  | 2041-2050 | BMR-SM-MELA-STK-NBP 3- RCP-8.5-2041-50 |
|  |  |  | 2051-2060 | BMR-SM-MELA-STK-NBP 3- RCP-8.5-2051-60 |
|  |  |  | 2061-2070 | BMR-SM-MELA-STK-NBP 3- RCP-8.5-2061-70 |
|  | NBP 4 | GFDL RCP-4.5 | 2031-2040 | BMR-SM-MELA-STK-NBP 4- RCP-4.5-2031-40 |
|  |  |  | 2041-2050 | BMR-SM-MELA-STK-NBP 4- RCP-4.5-2041-50 |
|  |  |  | 2051-2060 | BMR-SM-MELA-STK-NBP 4- RCP-4.5-2051-60 |
|  |  |  | 2061-2070 | BMR-SM-MELA-STK-NBP 4- RCP-4.5-2061-70 |
|  |  | GFDL RCP-8.5 | 2031-2040 | BMR-SM-MELA-STK-NBP 4- RCP-8.5-2031-40 |
|  |  |  | 2041-2050 | BMR-SM-MELA-STK-NBP 4- RCP-8.5-2041-50 |
|  |  |  | 2051-2060 | BMR-SM-MELA-STK-NBP 4- RCP-8.5-2051-60 |
|  |  |  | 2061-2070 | BMR-SM-MELA-STK-NBP 4- RCP-8.5-2061-70 |
|  | NBP 5 | GFDL RCP-4.5 | 2031-2040 | BMR-SM-MELA-STK-NBP 5- RCP-4.5-2031-40 |
|  |  |  | 2041-2050 | BMR-SM-MELA-STK-NBP 5- RCP-4.5-2041-50 |
|  |  |  | 2051-2060 | BMR-SM-MELA-STK-NBP 5- RCP-4.5-2051-60 |
|  |  |  | 2061-2070 | BMR-SM-MELA-STK-NBP 5- RCP-4.5-2061-70 |
|  |  | GFDL RCP-8.5 | 2031-2040 | BMR-SM-MELA-STK-NBP 5- RCP-8.5-2031-40 |
|  |  |  | 2041-2050 | BMR-SM-MELA-STK-NBP 5- RCP-8.5-2041-50 |
|  |  |  | 2051-2060 | BMR-SM-MELA-STK-NBP 5- RCP-8.5-2051-60 |
|  |  |  | 2061-2070 | BMR-SM-MELA-STK-NBP 5- RCP-8.5-2061-70 |

Appendix-A 7. Mann-Whitney U test results for Catchment management strategy and Biomass removal and Stand management attribute. For all the modelled response variables, P-value < 0.05. C represents the period from 1990–2015, as same as the baseline scenario’s period, R1 represents attribute + RCP–4.5 from 2031–2070, and R2 shows attribute + RCP–8.5 from 2031–2070.

| Catchment management strategy | | | | | | | | | | | | | | | | | | | | | | | | | |
| --- | --- | --- | --- | --- | --- | --- | --- | --- | --- | --- | --- | --- | --- | --- | --- | --- | --- | --- | --- | --- | --- | --- | --- | --- | --- |
| Flow, Nutrients and SS | NBP 1 | | | | | NBP 2 | | | | | NBP 3 | | | | | NBP 4 | | | | | NBP 5 | | | | |
|  | Sustain-  ability  first | | | | | Conventional  first | | | | | Self-sufficiency  first | | | | | City  first | | | | | Growth  first | | | | |
|  | C | | R1 | R2 | | C | | R1 | R2 | | C | | R1 | | R2 | C | | R1 | | R2 | C | | R1 | | R2 |
| Flow | U = -1094, N1 = N2 =26 | | U = -3916, N1 = N2 =50 | U = -3907, N1 = N2 =50 | | U = -1083, N1 = N2 =26 | | U = -3902, N1 = N2 =50 | U = -3898.5, N1 = N2 =50 | | U = -1029, N1 = N2 =26 | | U = -3791, N1 = N2 =50 | | U = -3776, N1 = N2 =50 | U = -1029, N1 = N2 =26 | | U = -3791, N1 = N2 =50 | | U = -3776, N1 = N2 =50 | U = -1029, N1 = N2 =26 | | U = -3791, N1 = N2 =50 | | U = -3776, N1 = N2 =50 |
| SS | U = -1114, N1 = N2 =26 | | U = -4034.5, N1 = N2 =50 | U = -4063, N1 = N2 =50 | | U = -1082, N1 = N2 =26 | | U = -3951, N1 = N2 =50 | U = -3982, N1 = N2 =50 | | U = -1035, N1 = N2 =26 | | U = -3786.5, N1 = N2 =50 | | U = -3789, N1 = N2 =50 | U = -1035, N1 = N2 =26 | | U = -3786.5, N1 = N2 =50 | | U = -3776, N1 = N2 =50 | U = -1035, N1 = N2 =26 | | U = -3786.5, N1 = N2 =50 | | U = -3814, N1 = N2 =50 |
| TN | U = -1015, N1 = N2 =26 | | U = -3767, N1 = N2 =50 | U = -3779, N1 = N2 =50 | | U = -1015, N1 = N2 =26 | | U = -3755, N1 = N2 =50 | U = -3784.5, N1 = N2 =50 | | U = -1060, N1 = N2 =26 | | U = -3755, N1 = N2 =50 | | U = -3770.5, N1 = N2 =50 | U = -1067, N1 = N2 =26 | | U = -3773, N1 = N2 =50 | | U = -3770.5, N1 = N2 =50 | U = -1047, N1 = N2 =26 | | U = -3755, N1 = N2 =50 | | U = -3770.5, N1 = N2 =50 |
| TP | U = -1037, N1 = N2 =26 | | U = -5952.25, N1 = N2 =50 | U = -6191, N1 = N2 =50 | | U = -1036.5, N1 = N2 =26 | | U = -5909, N1 = N2 =50 | U = -6141.5, N1 = N2 =50 | | U = -1036.5, N1 = N2 =26 | | U = -5800, N1 = N2 =50 | | U = -6049.5, N1 = N2 =50 | U = -1040, N1 = N2 =26 | | U = -5800, N1 = N2 =50 | | U = -6049.5, N1 = N2 =50 | U = -1036.5, N1 = N2 =26 | | U = -5800, N1 = N2 =50 | | U = -6049.5, N1 = N2 =50 |
| Org-N | U = -1024, N1 = N2 =26 | | U = -3832, N1 = N2 =50 | U = -3841, N1 = N2 =50 | | U = -1024, N1 = N2 =26 | | U = -3760, N1 = N2 =50 | U = -3770, N1 = N2 =50 | | U = -1038, N1 = N2 =26 | | U = -3724, N1 = N2 =50 | | U = -3731, N1 = N2 =50 | U = -1052, N1 = N2 =26 | | U = -3747, N1 = N2 =50 | | U = -3749, N1 = N2 =50 | U = -1031, N1 = N2 =26 | | U = -3721, N1 = N2 =50 | | U = -3731, N1 = N2 =50 |
| Org-P | U = -1053, N1 = N2 =26 | | U = -3846, N1 = N2 =50 | U = -3863, N1 = N2 =50 | | U = -1053, N1 = N2 =26 | | U = -3800, N1 = N2 =50 | U = -3821, N1 = N2 =50 | | U = -1061, N1 = N2 =26 | | U = -3766, N1 = N2 =50 | | U = -3814, N1 = N2 =50 | U = -1075, N1 = N2 =26 | | U = -3766, N1 = N2 =50 | | U = -3814, N1 = N2 =50 | U = -1053, N1 = N2 =26 | | U = -3766, N1 = N2 =50 | | U = -3814, N1 = N2 =50 |
| Biomass removal and Stand management | | | | | | | | | | | | | | | | | | | | | | | | | |
| Flow,  Nutrients  and SS | | NBP 1 | | | | | NBP 2 | | | | | NBP 3 | | | | | NBP 4 | | | | | NBP 5 | | | |
|  |  | R1 | | | R2 | | R1 | | | R2 | | R1 | | R2 | | | R1 | | R2 | | | R1 | | R2 | |
| Flow | | U = -193.76, N1 = N2 =10 | | | U = -211.72, N1 = N2 =10 | | U = -193.756, N1 = N2 =10 | | | U = -211.71, N1 = N2 =10 | | U = -198.95, N1 = N2 =10 | | U = -217.26, N1 = N2 =10 | | | U = -197.34, N1 = N2 =10 | | U = -215.56, N1 = N2 =10 | | | U = -299.13, N1 = N2 =10 | | U = -317.45, N1 = N2 =10 | |
| SS | | U = -174.23, N1 = N2 =10 | | | U = -191.47, N1 = N2 =10 | | U = -174.21, N1 = N2 =10 | | | U = -191.45, N1 = N2 =10 | | U = -178.31, N1 = N2 =10 | | U = -196.69, N1 = N2 =10 | | | U = -177.18, N1 = N2 =10 | | U = -195.19, N1 = N2 =10 | | | U = -278.32, N1 = N2 =10 | | U = -296.75, N1 = N2 =10 | |
| TN | | U = -646.81, N1 = N2 =10 | | | U = -689.25, N1 = N2 =10 | | U = -646.65, N1 = N2 =10 | | | U = -689.06, N1 = N2 =10 | | U = -754.62, N1 = N2 =10 | | U = -851.03, N1 = N2 =10 | | | U = -722.63, N1 = N2 =10 | | U = -797.97, N1 = N2 =10 | | | U = -859.18, N1 = N2 =10 | | U = -959.26, N1 = N2 =10 | |
| TP | | U = -174.23, N1 = N2 =10 | | | U = -191.47, N1 = N2 =10 | | U = -174.21, N1 = N2 =10 | | | U = -191.44, N1 = N2 =10 | | U = -178.31, N1 = N2 =10 | | U = -196.70, N1 = N2 =10 | | | U = -177.18, N1 = N2 =10 | | U = -195.19, N1 = N2 =10 | | | U = -278.32, N1 = N2 =10 | | U = -296.75, N1 = N2 =10 | |
| Org-N | | U = -448.33, N1 = N2 =10 | | | U = -528.76, N1 = N2 =10 | | U = -448.03, N1 = N2 =10 | | | U = -520.36, N1 = N2 =10 | | U = -507.20, N1 = N2 =10 | | U = -606.70, N1 = N2 =10 | | | U = -489.83, N1 = N2 =10 | | U = -581.67, N1 = N2 =10 | | | U = -518.85, N1 = N2 =10 | | U = -620.92, N1 = N2 =10 | |
| Org-P | | U = -646.81, N1 = N2 =10 | | | U = -689.25, N1 = N2 =10 | | U = -646.66, N1 = N2 =10 | | | U = -689.06, N1 = N2 =10 | | U = -754.62, N1 = N2 =10 | | U = -851.03, N1 = N2 =10 | | | U = -722.65, N1 = N2 =10 | | U = -797.97, N1 = N2 =10 | | | U = -859.18, N1 = N2 =10 | | U = -959.26, N1 = N2 =10 | |
