# Supplementary material for: Impacts on water quality in the peatland dominated catchment due to foreseen changes in Nordic Bioeconomy Pathways
Source: Sci Rep. 2023 Apr 18;13:6283. doi: 10.1038/s41598-023-33378-7 (PMC10113390; doi:10.1038/s41598-023-33378-7)
Supplement: Supplementary file 2 — Supplementary Information 2. [file 41598_2023_33378_MOESM2_ESM.docx]

# Supplementary Materials (SPM)

Impacts on water quality in the peatland dominated catchment due to foreseen changes in Nordic Bioeconomy Pathways

Joy Bhattacharjee^1*^, Hannu Marttila^1^, Eugenio Molina Navarro^2^, Artti Juutinen^3^, Anne Tolvanen^3^, Arto Haara^4^, Jouni Karhu^3^ and Bjørn Kløve^1^

^1^Water, Energy and Environmental Engineering Research Unit, PO Box 4300, 90014 University of Oulu, Oulu, Finland.

^2^Geology, Geography and Environment Department, University of Alcalá. Ctra. Madrid-Barcelona Km. 33.6, 28805, Alcalá de Henares, Madrid, Spain.

^3^Natural Resources Institute Finland (LUKE), Oulu, Finland.

^4^Natural Resources Institute Finland (LUKE), Joensuu, Finland.

*Corresponding author at Water, Energy and Environmental Engineering Research Unit, PO Box 4300, 90014 University of Oulu, Oulu, Finland. E-mail: joy.bhattacharjee@oulu.fi

## SPM-A: Forestry attributes

SPM-A 1. Detailed descriptions of the forestry attributes

| No. | **Forestry attributes** | **Explanation** |
| --- | --- | --- |
| 1 | **Dominant tree species: from novel monoculture to mixed natural** | **Novel monoculture: single-species stands of non-native exotic conifers (e.g., Sitka spruce, Contorta pine) or deciduous (e.g., poplar clones)**  **Middle: conventional mix, Norway spruce, Scots pine, and birch spp. in single or mixed-species stands**  **Mixed natural: local broadleaves (e.g., oak, beech, lime) and/or local coniferous trees in mixed-species stands** |
| 2 | **Stand management: from intensified biomass to nature-based** | **Intensified biomass: higher planting density (e.g., of spruce in conifer stands) and reduced thinning focused on maximising biomass volume**  **Middle: even-aged stand with multiple thinnings followed by clear-felling; managed primarily for timber value**  **Nature-based: uneven-aged stands, continuous cover forestry, and a focus on non-timber or non-biomass values such as biodiversity and other ecosystem services** |
| 3 | **Biomass removal at harvest: from intensified to reduced** | **Intensified: greater removal of needles, branches, and stumps primarily for energy production, harvesting of riparian and other currently protected areas**  **Middle: stem only harvesting with forest residues (needles, leaves, and branches) left on site, current protection of riparian and other sensitive areas**  **Reduced: selective harvesting and a focus on non-timber or non-biomass values such as biodiversity and other ecosystem services** |
| 4 | **Catchment management strategy: from production forests where possible to reduce** | **Production forests where possible: if the land can be used for forest products, it is; there is no consideration of sensitive areas/soils, water bodies, biodiversity, etc.**  **Middle: there is production in some sensitive areas but also an interest in protecting the most vulnerable sites**  **Protection of sensitive areas: no production on any sensitive area, protection of water bodies and sensitive soils, consideration of biodiversity** |
| 5 | **Fertiliser use: from intensified to none** | **Intensified: greater rates of nutrient input, either as mineral nitrogen fertiliser or as organic residues, more frequent fertiliser applications**  **Middle: single nitrogen fertiliser application late in the rotation to maximise harvestable timber value, current ash return practices**  **None: no inputs of nutrients, base cations (i.e., ash return), or other elements** |
| 6 | **Land cover: from increased agricultural land to increased forest land** | **Increased agricultural land: more land converted to agricultural production**  **Middle: current land cover proportions**  **Increased forest land: more land converted to forest production** |

**SPM-B: MELA: Structure and Processing**


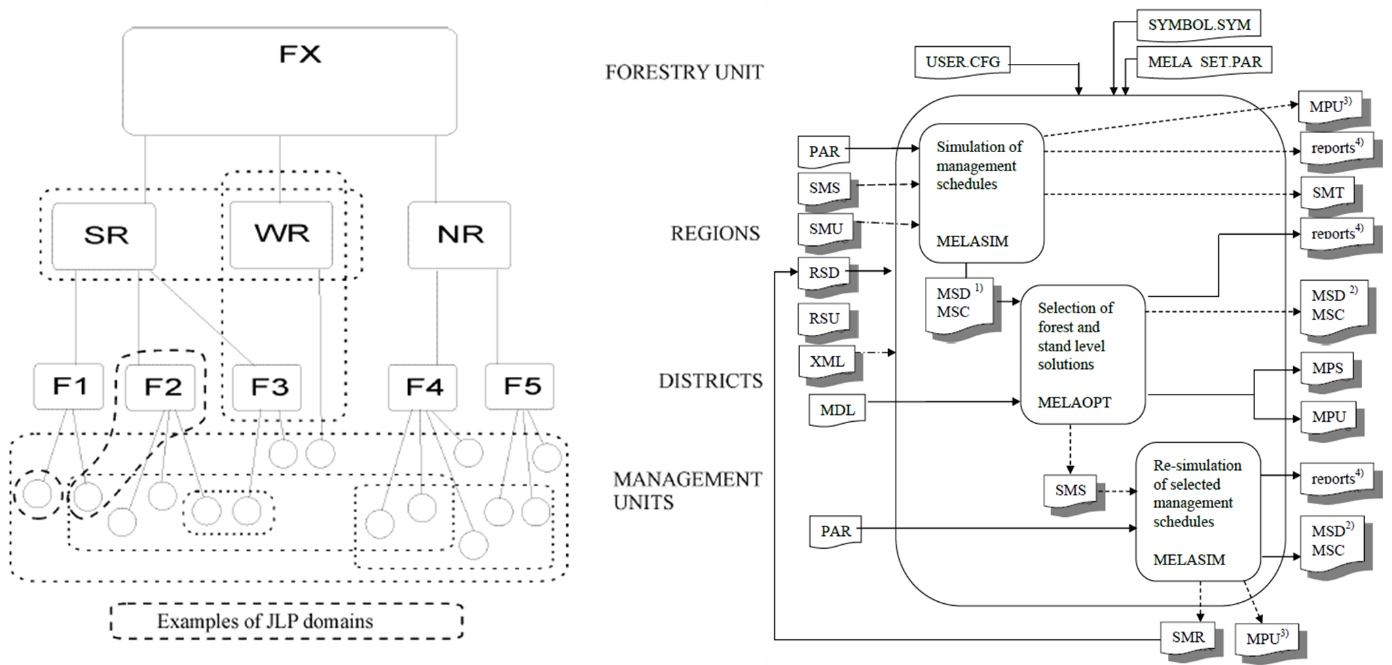


SPM-B 1. MELA structure and process of generating output files in MELA


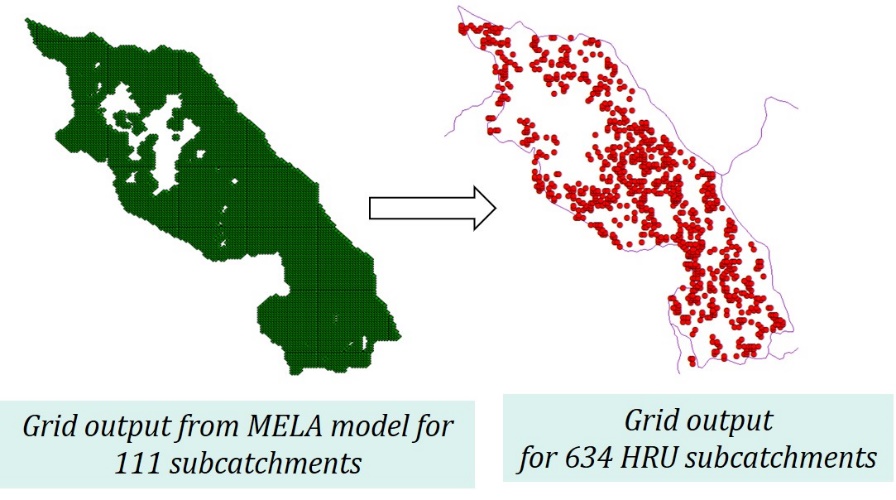


SPM-B 2. Distribution of MELA grid output from the sub-catchment to the HRU

**SPM-C: Python scripts and detailed steps to process the MELA output for use in SWAT.**

In the MELA grid output (.txt) in the Hydrologic Response Unit (HRU) database multiple steps were followed.

- First, all column variables from MELA output files were identified
- Point shapefiles were then created based on the grid coordinates for the entire catchment.
  - Subsequently, clipping was performed for points within each sub-catchment based on the 111 shapefiles of the sub-catchments.
  - A Python script (Step-I: Process of MELA data.py) was developed to consider all MELA output text files, merge them with the database of all subcatchments (111), and save csv format files with all the necessary columns for each decade.
  - Another Python script (Step-II: Conversion from Step-I csv output to dbf.py) was used to create a dbf file for each sub-catchment.
  - The old attributes of all the dbf files were replaced by the newly created dbf in the shapefile folder.
  - The same approach was applied to each HRU. Using another Python script (Step-III: Process of the MELA grid shape for each HRU of model.py), 634 point/grid files were created for each HRU.
- Thus, for the attributes SM, BMR, and FU, shapefiles were created for each NBP; however, each contained 188 columns. Thus, in total there were 3170 (634 based on HRU* 5 NBPs) shapefiles for each NBP.
- MELA simulates the results for each 10 years interval over 40 years. For this reason, for each variable, there were exactly four columns that represent 10 years average value of that specific variable for a different decade. Thus, the output values of the MELA variable represent different periods–2031-2040, 41-50, 51-60, and 61-70.
- Python scripts (as mentioned above) were applied again to separate the individual columns representing each decade for each HRU and NBP.
- At this stage, each HRU contains multiple points. However, according to the structure of the SWAT model, it was not feasible to integrate multiple points into the model. Thus, another Python script (Step-IV: Process of central feature (spatial average).py) was used from the grids available for each HRU.
- Next, all the central feature shapefiles were combined into one shapefile for each decade to provide it to the SWAT.
- Finally, for the BMR, SM, and FU attributes, the corresponding variables from the MELA model were selected from the central feature shapefile and assigned spatially to the management and plant database files of the SWAT model (mainly in the mgt1/mgt2/crop/plant file).

### Step-I: Processing of MELA data.py

#Importing necessary libraries

**import** os

**import** pandas **as** pd

**import** numpy **as** np

**import** dbf

**from** dbfread **import** DBF

#Loading all MELA output text files

**def** ListFilesInFolder**(**folder**):**

list_of_files **=** **[**os**.**path**.**join**(**folder**,**fn**)** **for** fn **in** **next(**os**.**walk**(**folder**))[**2**]** **if** fn**[-**4**:]** **==** '.txt'**]**

**return** list_of_files

#Ask user for the path to the input folder

input_folder**=** **input(**"Please specify full path to your input folder: "**)**

#Call the function ListFileInFolder and assign it to variable Myfiles

Myfiles**=**ListFilesInFolder**(**input_folder**)**

NBP_1 **=** pd**.**read_csv**(**Myfiles**[**0**],**sep**=**' '**).**sort_values**(**by**=**"vid"**)**

NBP_2 **=** pd**.**read_csv**(**Myfiles**[**4**],**sep**=**' '**).**sort_values**(**by**=**"vid"**)**

NBP_3 **=** pd**.**read_csv**(**Myfiles**[**3**],**sep**=**' '**).**sort_values**(**by**=**"vid"**)**

NBP_4 **=** pd**.**read_csv**(**Myfiles**[**1**],**sep**=**' '**).**sort_values**(**by**=**"vid"**)**

NBP_5 **=** pd**.**read_csv**(**Myfiles**[**2**],**sep**=**' '**).**sort_values**(**by**=**"vid"**)**

# Please change the column names based on the NBP scenario.

# NBP1 – metsa_ei.txt

# NBP2 – metsa_th.txt

# NBP3 – metsa_sske.txt

# NBP4 – metsa_jk.txt

# NBP5 – metsa_npv3.txt

#Loading all MELA output text files

**def** ListFilesInFolder**(**folder**):**

list_of_files **=** **[**os**.**path**.**join**(**folder**,**fn**)** **for** fn **in** **next(**os**.**walk**(**folder**))[**2**]** **if** fn**[-**4**:]** **==** '.dbf'**]**

**return** list_of_files

#Ask user for the path to the input folder

input_folder_dbf**=** **input(**"Please specify full path to your input folder: "**)**

#Call the function ListFileInFolder and assign it to variable Myfiles

Myfiles_dbf**=**ListFilesInFolder**(**input_folder_dbf**)**

Myfiles_dbf**.**sort**()**

**for** i **in** **range(len(**Myfiles_dbf**)):**

table **=** DBF**(**Myfiles_dbf**[**i**])**

a **=** **list(**table**)**

df_a **=** pd**.**DataFrame**(**a**)**

df_a**.**rename**(**columns**={**"Vid_from_m"**:**'vid'**},** inplace**=True)**

df_a**.**drop**(**df_a**.**columns**[**7**:**22**],** inplace**=True,** axis**=**1**)**

var_1 **=** NBP_5**[**NBP_5**[**"vid"**].**isin**(**df_a**[**"vid"**])]**

join_for_both **=** pd**.**merge**(**df_a**,**var_1**,**on **=** "vid"**,** how **=**'inner'**)**

list_0_mean **=** **[]**

list_1_mean **=** **[]**

list_2_mean **=** **[]**

list_3_mean **=** **[]**

list_4_mean **=** **[]**

**for** j **in** **range(len(**join_for_both**.**columns**)):**

**if** join_for_both**.**columns**[**j**][-**6**:]** **==** '0_MEAN'**:**

list_0_mean**.**append**(**join_for_both**.**columns**[**j**])**

**elif** join_for_both**.**columns**[**j**][-**6**:]** **==** '1_MEAN'**:**

list_1_mean**.**append**(**join_for_both**.**columns**[**j**])**

**elif** join_for_both**.**columns**[**j**][-**6**:]** **==** '2_MEAN'**:**

list_2_mean**.**append**(**join_for_both**.**columns**[**j**])**

**elif** join_for_both**.**columns**[**j**][-**6**:]** **==** '3_MEAN'**:**

list_3_mean**.**append**(**join_for_both**.**columns**[**j**])**

**elif** join_for_both**.**columns**[**j**][-**6**:]** **==** '4_MEAN'**:**

list_4_mean**.**append**(**join_for_both**.**columns**[**j**])**

**del** list_1_mean**[**4**:**8**]**

for_all_period_based_columns **=** list_0_mean **+** list_1_mean **+** list_2_mean **+** list_3_mean **+** list_4_mean

list_0_mean_columns **=** join_for_both**[**list_0_mean**]**

list_1_mean_columns **=** join_for_both**[**list_1_mean**]**

list_2_mean_columns **=** join_for_both**[**list_2_mean**]**

list_3_mean_columns **=** join_for_both**[**list_3_mean**]**

list_4_mean_columns **=** join_for_both**[**list_4_mean**]**

####################################################################

listed_columns **=** join_for_both**.**drop**(**columns **=** for_all_period_based_columns**)**

list_F1A **=** **[]**

list_F1L **=** **[]**

list_F2L **=** **[]**

list_F3L **=** **[]**

list_F4L **=** **[]**

**for** k **in** **range(len(**listed_columns**.**columns**)):**

**if** listed_columns**.**columns**[**k**][**11**:**14**]** **==** "F1A"**:**

list_F1A**.**append**(**listed_columns**.**columns**[**k**])**

**elif** listed_columns**.**columns**[**k**][**11**:**14**]** **==** "F1L"**:**

list_F1L**.**append**(**listed_columns**.**columns**[**k**])**

**elif** listed_columns**.**columns**[**k**][**11**:**14**]** **==** "F2L"**:**

list_F2L**.**append**(**listed_columns**.**columns**[**k**])**

**elif** listed_columns**.**columns**[**k**][**11**:**14**]** **==** "F3L"**:**

list_F3L**.**append**(**listed_columns**.**columns**[**k**])**

**elif** listed_columns**.**columns**[**k**][**11**:**14**]** **==** "F4L"**:**

list_F4L**.**append**(**listed_columns**.**columns**[**k**])**

for_all_F_based_L_columns **=** list_F1L **+** list_F2L **+** list_F3L **+** list_F4L

list_F1L_columns **=** listed_columns**[**list_F1L**]**

list_F2L_columns **=** listed_columns**[**list_F2L**]**

list_F3L_columns **=** listed_columns**[**list_F3L**]**

list_F4L_columns **=** listed_columns**[**list_F4L**]**

#change column name here

list_F2L_columns**[**"metsa_npv3_F2L_Vkok_MEAN"**]** **=** list_F1L_columns**.**metsa_npv3_F1L_Vkok_1_MEAN

list_F1L_columns**.**metsa_npv3_F1L_HPKu_MEAN **=** list_F1L_columns**.**metsa_npv3_F1L_HPku_1_MEAN

list_F1L_columns**.**metsa_npv3_F1L_HPTlpuu_MEAN **=** list_F1L_columns**.**metsa_npv3_F1L_HPlp_1_MEAN

list_F1L_columns**.**metsa_npv3_F1L_HPman_MEAN **=** list_F1L_columns**.**metsa_npv3_F1L_HPma_1_MEAN

list_F1L_columns **=** list_F1L_columns**.**drop**(**columns **=** **[**"metsa_npv3_F1L_Vkok_1_MEAN"**,**"metsa_npv3_F1L_HPku_1_MEAN"**,**

"metsa_npv3_F1L_HPlp_1_MEAN"**,** "metsa_npv3_F1L_HPma_1_MEAN"**,**

"metsa_npv3_F1L_yht_MEAN"**])**

list_F2L_columns**.**columns **=** list_F1L_columns**.**columns

list_F3L_columns**.**columns **=** list_F1L_columns**.**columns

list_F4L_columns**.**columns **=** list_F1L_columns**.**columns

df_for_F_average **=** pd**.**concat**([**list_F1L_columns**,** list_F2L_columns**,** list_F3L_columns**,** list_F4L_columns**],** axis **=** 0**).**groupby**(**level**=**0**).**mean**()**

Columns_common_for_all_except_decade **=** listed_columns**.**drop**(**columns **=** for_all_F_based_L_columns**)**

Column_for_0_decade **=** pd**.**concat**([**Columns_common_for_all_except_decade**,** list_0_mean_columns**,** df_for_F_average**],** axis **=** 1**)**

Column_for_0_decade**.**columns **=** **[**r**[-**10**:]** **for** r **in** Column_for_0_decade**.**columns**]**

#########################

Column_for_1_decade **=** pd**.**concat**([**Columns_common_for_all_except_decade**,** list_1_mean_columns**,** df_for_F_average**],** axis **=** 1**)**

Column_for_1_decade**.**columns **=** **[**r**[-**10**:]** **for** r **in** Column_for_1_decade**.**columns**]**

Column_for_2_decade **=** pd**.**concat**([**Columns_common_for_all_except_decade**,** list_2_mean_columns**,** df_for_F_average**],** axis **=** 1**)**

Column_for_2_decade**.**columns **=** **[**r**[-**10**:]** **for** r **in** Column_for_2_decade**.**columns**]**

Column_for_3_decade **=** pd**.**concat**([**Columns_common_for_all_except_decade**,** list_3_mean_columns**,** df_for_F_average**],** axis **=** 1**)**

Column_for_3_decade**.**columns **=** **[**r**[-**10**:]** **for** r **in** Column_for_3_decade**.**columns**]**

Column_for_4_decade **=** pd**.**concat**([**Columns_common_for_all_except_decade**,** list_4_mean_columns**,** df_for_F_average**],** axis **=** 1**)**

Column_for_4_decade**.**columns **=** **[**r**[-**10**:]** **for** r **in** Column_for_4_decade**.**columns**]**

#Creating dataframe for each decade

# Please specify your output folder where you want to save dbf within the os.chdir command

name **=** Myfiles_dbf**[**i**].**split**(**"\\"**)[-**1**].**split**(**"."**)[**0**]**

os**.**chdir**(**r"folder location"**)**

Column_for_0_decade**.**to_csv**(str(**name**)** **+** '.csv'**,** sep**=**','**,** header**=True,** index **=** **False,** float_format**=**'%.10f'**,** mode**=**'w'**)**

os**.**chdir**(**r"folder location"**)**

Column_for_1_decade**.**to_csv**(str(**name**)** **+** '.csv'**,** sep**=**','**,** header**=True,** index **=** **False,** float_format**=**'%.10f'**,** mode**=**'w'**)**

os**.**chdir**(**r"folder location"**)**

Column_for_2_decade**.**to_csv**(str(**name**)** **+** '.csv'**,** sep**=**','**,** header**=True,** index **=** **False,** float_format**=**'%.10f'**,** mode**=**'w'**)**

os**.**chdir**(**r"folder location"**)**

Column_for_3_decade**.**to_csv**(str(**name**)** **+** '.csv'**,** sep**=**','**,** header**=True,** index **=** **False,** float_format**=**'%.10f'**,** mode**=**'w'**)**

os**.**chdir**(**r"folder location"**)**

Column_for_4_decade**.**to_csv**(str(**name**)** **+** '.csv'**,** sep**=**','**,** header**=True,** index **=** **False,** float_format**=**'%.10f'**,** mode**=**'w'**)**

### Step-II: Conversion from step-1 csv output to dbf.py

# importing modules

**import** os

**import** arcpy

**from** arcpy **import** env

arcpy**.**env**.**overwriteOutput**=True**

#Please put your default gdb

env**.**workspace **=** r"Provide your environment workspace"

**def** ListFilesInFolder**(**folder**):**

list_of_files **=** **[**os**.**path**.**join**(**folder**,**fn**)** **for** fn **in** **next(**os**.**walk**(**folder**))[**2**]** **if** fn**[-**4**:]** **==** '.csv'**]**

**return** list_of_files

#Ask user for the path to the input folder

input_folder**=** **input(**r"Please specify full path to your input folder: "**)**

Myfiles**=**ListFilesInFolder**(**input_folder**)**

out_folder**=** r"Please specify full path to your output folder: "

**for** i **in** Myfiles**:**

arcpy**.**TableToDBASE_conversion**(**i**,**out_folder**)**

### Step-III: Process of MELA grid shape for each HRU of the model.py

# importing modules

**import** os

**import** arcpy

**from** arcpy **import** env

arcpy**.**env**.**overwriteOutput**=True**

#Please provide your default gdb

env**.**workspace **=** r"Provide your environment workspace"

**def** ListFilesInFolder**(**folder**):**

list_of_files **=** **[**os**.**path**.**join**(**folder**,**fn**)** **for** fn **in** **next(**os**.**walk**(**folder**))[**2**]** **if** fn**[-**4**:]** **==** '.shp'**]**

**return** list_of_files

#Ask user for the path to the input folder

MELA_30_sub **=** ListFilesInFolder**(**r"Please specify full path to your input folder:"**)**

HRU_folder **=** ListFilesInFolder **(**r"Please specify full path to your input folder:"**)**

**for** i **in** **range(len(**MELA_30_sub**)):**

**for** j **in** **range(len(**HRU_folder**)):**

**if** **(**i **>** 70**):**

**if** MELA_30_sub**[**i**].**split**(**"\\"**)[-**1**].**split**(**"."**)[**0**]** **==** HRU_folder**[**j**].**split**(**"\\"**)[-**1**].**split**(**"."**)[**0**].**split**(**"_"**)[**0**]:**

**print** **(**MELA_30_sub**[**i**].**split**(**"\\"**)[-**1**],** HRU_folder**[**j**].**split**(**"\\"**)[-**1**],**i**,**j**)**

out_feature_class **=** HRU_folder**[**j**].**split**(**"\\"**)[-**1**]**

arcpy**.**Clip_analysis**(**MELA_30_sub**[**i**],** HRU_folder**[**j**],** out_feature_class**)**

### Step-IV: Step-4: Process of central feature (spatial average).py

**import** os

**import** arcpy

**from** arcpy **import** env

arcpy**.**env**.**overwriteOutput**=True**

#Please provide your default gdb

env**.**workspace **=** r"Provide your environment workspace"

**def** ListFilesInFolder**(**folder**):**

list_of_files **=** **[**os**.**path**.**join**(**folder**,**fn**)** **for** fn **in** **next(**os**.**walk**(**folder**))[**2**]** **if** fn**[-**4**:]** **==** '.shp'**]**

**return** list_of_files

#Ask user for the path to the input folder

input_folder**=** **input** (r"Please specify full path to your input folder:"**)**

Myfiles**=**ListFilesInFolder**(**input_folder**)**

out_folder**=** r"Please specify full path to your output folder: "

**for** i **in** **range(len(**Myfiles**)):**

out_feature_class **=** Myfiles**[**i**].**split**(**"\\"**)[-**1**]**

arcpy**.**CentralFeature_stats**(**Myfiles**[**i**],** out_feature_class**,** "MANHATTAN_DISTANCE"**)**

## SPM-D: Comparison of Climate model with historical data


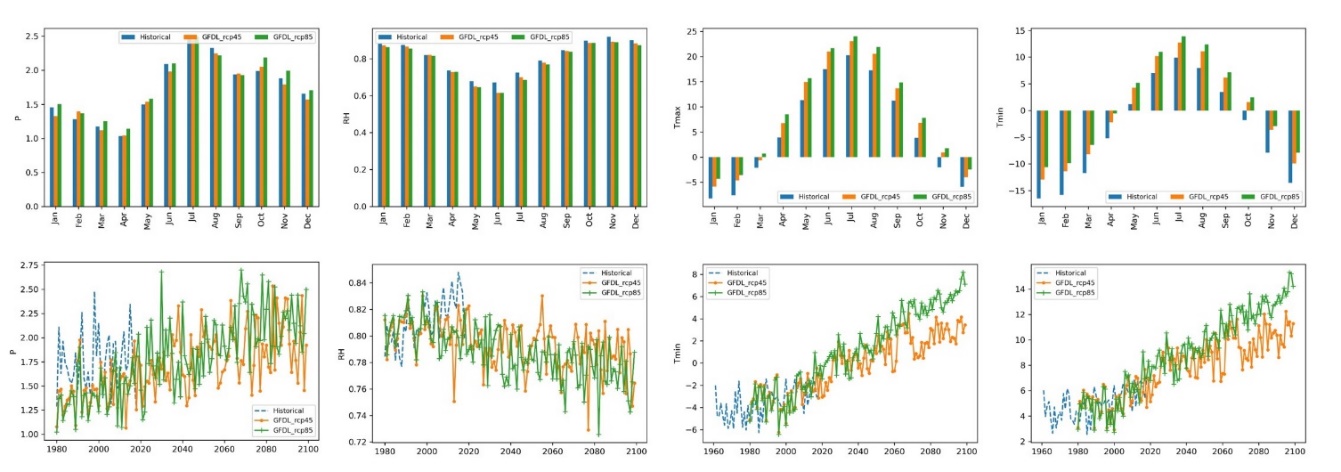


SPM-D 1. Monthly and yearly comparisons of the Regional climate model data (RCP 4.5, RCP 8.5) with historical data

**SPM-E: Final Python Scripts to process results of LSM attributes for NBPs**

### CMS attribute

# Importing all modules

**import** numpy **as** np

**import** itertools

**from** math **import** sqrt**,**ceil

**from** skimage **import** feature

**import** pandas **as** pd

**import** fnmatch

**import** csv

**import** shutil

**import** os

**import** matplotlib **as** plt

**import** numpy **as** np

**import** matplotlib**.**pyplot **as** plt

**from** matplotlib**.**ticker **import** NullFormatter

**from** datetime **import** datetime

**import** scipy**.**stats **as** st

**import** seaborn **as** sns

##################### Step-01: Loading Images, naming files #########################

## Function to list all tif files in the input folder with the path to folder as a variable

**def** ListFilesInFolder**(**pattern**,** folder**):**

list_of_files **=** **[]**

pattern **=** pattern

**for** path**,** subdirs**,** files **in** os**.**walk**(**folder**):**

**for** name **in** files**:**

**if** fnmatch**.**fnmatch**(**name**,** pattern**):**

list_of_files**.**append**(**os**.**path**.**join**(**path**,** name**))**

**return** list_of_files

input_folder**=** r"Provide folder location where output of CMS attributes are stored"

GFDL_45_inp_folder **=** r"Provide folder location where output of CMS attributes+GFDL_RCP_4.5 are stored"

GFDL_85_inp_folder **=** r"Provide folder location where output of CMS attributes+GFDL_RCP_8.5 are stored"

out_folder **=** r"Provide folder location where you want to save the output images"

## Call the function ListFileInFolder and assign it to the variable Myfiles

Flow_111**=**ListFilesInFolder**(**"R-Flow_*111.txt"**,** input_folder**)**

Flow_111_GFDL45 **=**ListFilesInFolder**(**"R-Flow_*111.txt"**,** GFDL_45_inp_folder**)**

Flow_111_GFDL85 **=**ListFilesInFolder**(**"R-Flow_*111.txt"**,** GFDL_85_inp_folder**)**

OrgN_111**=**ListFilesInFolder**(**"R-OrgN_*111.txt"**,** input_folder**)**

OrgN_111_GFDL45 **=** ListFilesInFolder**(**"R-OrgN_*111.txt"**,** GFDL_45_inp_folder**)**

OrgN_111_GFDL85 **=** ListFilesInFolder**(**"R-OrgN_*111.txt"**,** GFDL_85_inp_folder**)**

Tn_111**=** ListFilesInFolder**(**"*N_111.txt"**,** input_folder**)**

Tn_111_GFDL45 **=** ListFilesInFolder**(**"*N_111.txt"**,** GFDL_45_inp_folder**)**

Tn_111_GFDL85 **=** ListFilesInFolder**(**"*N_111.txt"**,** GFDL_85_inp_folder**)**

OrgP_111**=** ListFilesInFolder**(**"R-ORGP_*111.txt"**,** input_folder**)**

OrgP_111_GFDL45 **=** ListFilesInFolder**(**"R-OrgP_*111.txt"**,** GFDL_45_inp_folder**)**

OrgP_111_GFDL85 **=** ListFilesInFolder**(**"R-OrgP_*111.txt"**,** GFDL_85_inp_folder**)**

Tp_111**=**ListFilesInFolder**(**"*P_111.txt"**,** input_folder**)**

Tp_111_GFDL45 **=** ListFilesInFolder**(**"*P_111.txt"**,** GFDL_45_inp_folder**)**

Tp_111_GFDL85 **=** ListFilesInFolder**(**"*P_111.txt"**,** GFDL_85_inp_folder**)**

Sed_111**=**ListFilesInFolder**(**"R-Sed_*111.txt"**,** input_folder**)**

Sed_111_GFDL45 **=** ListFilesInFolder**(**"R-Sed_*111.txt"**,** GFDL_45_inp_folder**)**

Sed_111_GFDL85 **=** ListFilesInFolder**(**"R-Sed_*111.txt"**,** GFDL_85_inp_folder**)**

# Reading all measured data

Measure_date **=** pd**.**read_csv**(**r"Provide date of your measured data in a csv file"**,** sep**=**','**,** header**=**'infer'**)**

Measure_date**.**columns

os**.**chdir**(**out_folder**)**

Climate_date **=** pd**.**date_range**(**start**=**"2031/01/01"**,** end**=**"2070/12/31"**)**

Climate_date_without_obs **=** pd**.**date_range**(**start**=**"1990/01/01"**,** end**=**"2015/12/31"**)**

#################### Function #####################################

**def** plot**(**Var_file_list**,**Var_file_list_45**,** Var_file_list_85**,** Climate_date_without_obs**,** Climate_date**,** col_name**,** var**,** unit**):**

############################

list_of_loaded_data **=** **[]**

column_list_from_loaded_data **=** **[]**

**for** i **in** **range(len(**Var_file_list**)):**

data **=** np**.**loadtxt**(**Var_file_list**[**i**],** dtype**=** **(str),** delimiter**=**" "**,** skiprows **=** 1**)**

data_col **=** data**[:,**2**]**

list_of_loaded_data**.**append**(**data_col**)**

column_list_from_loaded_data**.**append**(**Var_file_list**[**i**].**split**(**"\\"**)[-**2**])**

list_of_loaded_data_45 **=** **[]**

column_list_from_loaded_data_45 **=** **[]**

**for** i **in** **range(len(**Var_file_list_45**)):**

data_45 **=** np**.**loadtxt**(**Var_file_list_45**[**i**],** dtype**=** **(str),** delimiter**=**" "**,** skiprows **=** 1**)**

data_col_45 **=** data_45**[:,**2**]**

list_of_loaded_data_45**.**append**(**data_col_45**)**

column_list_from_loaded_data_45**.**append**(**Var_file_list_45**[**i**].**split**(**"\\"**)[-**2**])**

list_of_loaded_data_85 **=** **[]**

column_list_from_loaded_data_85 **=** **[]**

**for** i **in** **range(len(**Var_file_list_85**)):**

data_85 **=** np**.**loadtxt**(**Var_file_list_85**[**i**],** dtype**=** **(str),** delimiter**=**" "**,** skiprows **=** 1**)**

data_col_85 **=** data_85**[:,**2**]**

list_of_loaded_data_85**.**append**(**data_col_85**)**

column_list_from_loaded_data_85**.**append**(**Var_file_list_85**[**i**].**split**(**"\\"**)[-**2**])**

df **=** pd**.**DataFrame**(**list_of_loaded_data**,**dtype**=float)**

df_transposed **=** df**.**T

df_transposed**.**columns **=** column_list_from_loaded_data

df_45 **=** pd**.**DataFrame**(**list_of_loaded_data_45**,**dtype**=float)**

df_transposed_45 **=** df_45**.**T

df_transposed_45**.**columns **=** column_list_from_loaded_data_45

df_85 **=** pd**.**DataFrame**(**list_of_loaded_data_85**,**dtype**=float)**

df_transposed_85 **=** df_85**.**T

df_transposed_85**.**columns **=** column_list_from_loaded_data_85

#####################################

Date_final_C **=** pd**.**DataFrame**(**Climate_date_without_obs**,** columns **=** **[**"Date"**])**

Date_final_C**[**'Year'**]** **=** Date_final_C**[**"Date"**].**dt**.**year

Date_final_C**[**'Month'**]** **=** Date_final_C**[**"Date"**].**dt**.**month

Date_final_C**.**index **=** Date_final_C**[**"Date"**]**

Date_final **=** pd**.**DataFrame**(**Climate_date**,** columns **=** **[**"Date"**])**

Date_final**[**'Year'**]** **=** Date_final**[**"Date"**].**dt**.**year

Date_final**[**'Month'**]** **=** Date_final**[**"Date"**].**dt**.**month

Date_final**.**index **=** Date_final**[**"Date"**]**

################################################

# Processing flow data and saving descriptions for each NBP

df_transposed**.**index **=** Date_final_C**[**"Date"**]**

df_transposed_45**.**index **=** Date_final**[**"Date"**]**

df_transposed_85**.**index **=** Date_final**[**"Date"**]**

# df_des = df_transposed.describe()

# per year

df_transposed_year **=** df_transposed**.**merge**(**Date_final_C**[**'Year'**],** left_index **=** **True,** right_index**=True)**

df_transposed_year_45 **=** df_transposed_45**.**merge**(**Date_final**[**'Year'**],** left_index **=** **True,** right_index**=True)**

df_transposed_year_85 **=** df_transposed_85**.**merge**(**Date_final**[**'Year'**],** left_index **=** **True,** right_index**=True)**

#########################################################

avg_df_transposed_year **=** df_transposed_year**.**groupby**([**'Year'**]).**mean**()**

avg_df_transposed_year_45 **=** df_transposed_year_45**.**groupby**([**'Year'**]).**mean**()**

avg_df_transposed_year_85 **=** df_transposed_year_85**.**groupby**([**'Year'**]).**mean**()**

total_df **=** pd**.**concat**([**avg_df_transposed_year**,**avg_df_transposed_year_45**,**avg_df_transposed_year_85**])**

total_df**.**to_csv**(**'%s.csv'**%(**col_name**),** sep**=**','**,** header**=True,** index**=True,** float_format**=**'%.3f'**,** mode**=**'w'**)**

# yearly changes from baseline processing

avg_df_transposed_year_minus_from_NBP0**=** avg_df_transposed_year**.**sub**(**avg_df_transposed_year **[**"NBP0"**],**axis **=**0**)**

avg_df_transposed_year_minus_from_NBP0_45**=** avg_df_transposed_year_45**.**sub**(**avg_df_transposed_year_45 **[**"NBP0"**],**axis **=**0**)**

avg_df_transposed_year_minus_from_NBP0_85**=** avg_df_transposed_year_85**.**sub**(**avg_df_transposed_year_85 **[**"NBP0"**],**axis **=**0**)**

# per year change

avg_df_transposed_year_change_percent_from_NBP0**=** **(**avg_df_transposed_year_minus_from_NBP0**.**div**(**avg_df_transposed_year **[**"NBP0"**]** **,**axis **=**0**))***100

avg_df_transposed_year_change_percent_from_NBP0_45**=** **(**avg_df_transposed_year_minus_from_NBP0_45**.**div**(**avg_df_transposed_year_45 **[**"NBP0"**]** **,**axis **=**0**))***100

avg_df_transposed_year_change_percent_from_NBP0_85**=** **(**avg_df_transposed_year_minus_from_NBP0_85**.**div**(**avg_df_transposed_year_85 **[**"NBP0"**]** **,**axis **=**0**))***100

# for boxplot

# per year

df_final_for_boxplot_per_year **=** avg_df_transposed_year_change_percent_from_NBP0**.**drop**(**columns **=** **[**"NBP0"**])**

df_final_for_boxplot_per_year **[**"Baseline"**]** **=**"Current period"

df_final_for_boxplot_per_year_45 **=** avg_df_transposed_year_change_percent_from_NBP0_45**.**drop**(**columns **=** **[**"NBP0"**])**

df_final_for_boxplot_per_year_45 **[**"Baseline"**]** **=**"RCP-4.5"

df_final_for_boxplot_per_year_85 **=** avg_df_transposed_year_change_percent_from_NBP0_85**.**drop**(**columns **=** **[**"NBP0"**])**

df_final_for_boxplot_per_year_85 **[**"Baseline"**]** **=**"RCP-8.5"

com_df **=** pd**.**concat**([**df_final_for_boxplot_per_year**,**df_final_for_boxplot_per_year_45**,**df_final_for_boxplot_per_year_85**])**

mod_df **=** pd**.**melt**(**com_df**,**id_vars**=**"Baseline"**)**

sns**.**boxplot**(**x **=**"variable"**,** y **=** "value"**,** hue **=**"Baseline"**,** data**=** mod_df**,** showfliers **=** **False,** palette **=**"muted"**)**

sns**.**despine**(**offset**=**1**,** trim **=True)**

plt**.**legend**(**title **=** ""**,** fontsize **=** 12**)**

plt**.**xlabel**(**''**)**

plt**.**ylabel**(**'Annual Change from NBP0 (%)'**,**fontsize **=** 12**)**

plt**.**legend**(**loc **=** 'lower right'**,** prop**={**'size'**:**8**},** ncol **=** 1**)**

plt**.**savefig **(str(**col_name**)+**' yearly_boxplot.jpg'**,** dpi **=** 300**)**

plt**.**show**()**

plt**.**close **()**

### Final output plot. An example of Flow plot. Same code is applicable for nutrients and SS ###

plot**(**Flow_111**,** Flow_111_GFDL45**,** Flow_111_GFDL85**,** Climate_date_without_obs**,** Climate_date**,** 'Simo_Flow'**,** " Flow "**,** "(m3/s)"**)**

### BMR-SM attribute

# Import all modules

**import** numpy **as** np

**import** itertools

**from** math **import** sqrt**,**ceil

**from** skimage **import** feature

**import** pandas **as** pd

**import** fnmatch

**import** csv

**import** shutil

**import** os

**import** matplotlib **as** plt

**import** numpy **as** np

**import** matplotlib**.**pyplot **as** plt

**from** matplotlib**.**ticker **import** NullFormatter

**from** datetime **import** datetime

**import** scipy**.**stats **as** st

**import** seaborn **as** sns

##################### Step-01: Loading Images, naming files #########################

## Function to list all tif files in the input folder with a path to folder as a variable

**def** ListFilesInFolder**(**pattern**,** folder**):**

list_of_files **=** **[]**

pattern **=** pattern

**for** path**,** synubdirs**,** files **in** os**.**walk**(**folder**):**

**for** name **in** files**:**

**if** fnmatch**.**fnmatch**(**name**,** pattern**):**

list_of_files**.**append**(**os**.**path**.**join**(**path**,** name**))**

**return** list_of_files

GFDL_45_inp_folder **=** r"Provide folder location of the output of Biomass + RCP-4.5"

GFDL_85_inp_folder **=** r"Provide folder location of the output of Biomass + RCP-8.5"

out_folder **=** r"Provide output folder location"

## Call the function ListFileInFolder and assign it to the variable Myfiles

Flow_111_GFDL45 **=**ListFilesInFolder**(**"R-Flow_*111.txt"**,** GFDL_45_inp_folder**)**

Flow_111_GFDL85 **=**ListFilesInFolder**(**"R-Flow_*111.txt"**,** GFDL_85_inp_folder**)**

OrgN_111_GFDL45 **=** ListFilesInFolder**(**"R-OrgN_*111.txt"**,** GFDL_45_inp_folder**)**

OrgN_111_GFDL85 **=** ListFilesInFolder**(**"R-OrgN_*111.txt"**,** GFDL_85_inp_folder**)**

Tn_111_GFDL45 **=** ListFilesInFolder**(**"*N_111.txt"**,** GFDL_45_inp_folder**)**

Tn_111_GFDL85 **=** ListFilesInFolder**(**"*N_111.txt"**,** GFDL_85_inp_folder**)**

OrgP_111_GFDL45 **=** ListFilesInFolder**(**"R-OrgP_*111.txt"**,** GFDL_45_inp_folder**)**

OrgP_111_GFDL85 **=** ListFilesInFolder**(**"R-OrgP_*111.txt"**,** GFDL_85_inp_folder**)**

Tp_111_GFDL45 **=** ListFilesInFolder**(**"*P_111.txt"**,** GFDL_45_inp_folder**)**

Tp_111_GFDL85 **=** ListFilesInFolder**(**"*P_111.txt"**,** GFDL_85_inp_folder**)**

Sed_111_GFDL45 **=** ListFilesInFolder**(**"R-Sed_*111.txt"**,** GFDL_45_inp_folder**)**

Sed_111_GFDL85 **=** ListFilesInFolder**(**"R-Sed_*111.txt"**,** GFDL_85_inp_folder**)**

Climate_date **=** pd**.**date_range**(**start**=**"2031/01/01"**,** end**=**"2070/12/31"**)**

os**.**chdir**(**out_folder**)**

#################### Function #####################################

**def** plot**(**Var_file_list**,** Measure_date**,** col_name**,** var**,** unit**,** X**,** Y **):**

############################

NBP_0 **=** **[]**

NBP_1 **=** **[]**

NBP_2 **=** **[]**

NBP_3 **=** **[]**

NBP_4 **=** **[]**

NBP_5 **=** **[]**

column_list_from_loaded_data **=** **[]**

**for** i **in** **range(len(**Var_file_list**)):**

**if** Var_file_list**[**i**].**split**(**"\\"**)[-**3**]** **==** 'NBP0'**:**

data **=** np**.**loadtxt**(**Var_file_list**[**i**],** dtype**=** **(str),** delimiter**=**" "**,** skiprows **=** 1**)**

data_col **=** data**[:,**2**]**

**for** j **in** data_col**:**

NBP_0**.**append**(**j**)**

column_list_from_loaded_data**.**append**(**Var_file_list**[**i**].**split**(**"\\"**)[-**3**]+**'_' **+** Var_file_list**[**i**].**split**(**"\\"**)[-**2**])**

**elif** Var_file_list**[**i**].**split**(**"\\"**)[-**3**]** **==** 'NBP1'**:**

data **=** np**.**loadtxt**(**Var_file_list**[**i**],** dtype**=** **(str),** delimiter**=**" "**,** skiprows **=** 1**)**

data_col **=** data**[:,**2**]**

**for** j **in** data_col**:**

NBP_1**.**append**(**j**)**

column_list_from_loaded_data**.**append**(**Var_file_list**[**i**].**split**(**"\\"**)[-**3**]+**'_' **+** Var_file_list**[**i**].**split**(**"\\"**)[-**2**])**

**elif** Var_file_list**[**i**].**split**(**"\\"**)[-**3**]** **==** 'NBP2'**:**

data **=** np**.**loadtxt**(**Var_file_list**[**i**],** dtype**=** **(str),** delimiter**=**" "**,** skiprows **=** 1**)**

data_col **=** data**[:,**2**]**

**for** j **in** data_col**:**

NBP_2**.**append**(**j**)**

column_list_from_loaded_data**.**append**(**Var_file_list**[**i**].**split**(**"\\"**)[-**3**]+**'_' **+** Var_file_list**[**i**].**split**(**"\\"**)[-**2**])**

**elif** Var_file_list**[**i**].**split**(**"\\"**)[-**3**]** **==** 'NBP3'**:**

data **=** np**.**loadtxt**(**Var_file_list**[**i**],** dtype**=** **(str),** delimiter**=**" "**,** skiprows **=** 1**)**

data_col **=** data**[:,**2**]**

**for** j **in** data_col**:**

NBP_3**.**append**(**j**)**

column_list_from_loaded_data**.**append**(**Var_file_list**[**i**].**split**(**"\\"**)[-**3**]+**'_' **+** Var_file_list**[**i**].**split**(**"\\"**)[-**2**])**

**elif** Var_file_list**[**i**].**split**(**"\\"**)[-**3**]** **==** 'NBP4'**:**

data **=** np**.**loadtxt**(**Var_file_list**[**i**],** dtype**=** **(str),** delimiter**=**" "**,** skiprows **=** 1**)**

data_col **=** data**[:,**2**]**

**for** j **in** data_col**:**

NBP_4**.**append**(**j**)**

column_list_from_loaded_data**.**append**(**Var_file_list**[**i**].**split**(**"\\"**)[-**3**]+**'_' **+** Var_file_list**[**i**].**split**(**"\\"**)[-**2**])**

**elif** Var_file_list**[**i**].**split**(**"\\"**)[-**3**]** **==** 'NBP5'**:**

data **=** np**.**loadtxt**(**Var_file_list**[**i**],** dtype**=** **(str),** delimiter**=**" "**,** skiprows **=** 1**)**

data_col **=** data**[:,**2**]**

**for** j **in** data_col**:**

NBP_5**.**append**(**j**)**

column_list_from_loaded_data**.**append**(**Var_file_list**[**i**].**split**(**"\\"**)[-**3**]+**'_' **+** Var_file_list**[**i**].**split**(**"\\"**)[-**2**])**

NBP_0 **=** NBP_0 **[**X**:**Y**]**

NBP_1 **=** NBP_1 **[**X**:**Y**]**

NBP_2 **=** NBP_2 **[**X**:**Y**]**

NBP_3 **=** NBP_3 **[**X**:**Y**]**

NBP_4 **=** NBP_4 **[**X**:**Y**]**

NBP_5 **=** NBP_5 **[**X**:**Y**]**

Measure_date **=** Measure_date **[**X**:**Y**]**

NBP_0 **=** pd**.**DataFrame**(**NBP_0**,**dtype**=float,** columns **=** **[**'NBP0'**])**

NBP_0**.**index **=** Measure_date

df_main **=** pd**.**DataFrame**(list(zip(**NBP_1**,**NBP_2**,**NBP_3**,**NBP_4**,**NBP_5**)),** dtype**=float,** columns **=** **[**'NBP1'**,**'NBP2'**,**'NBP3'**,**'NBP4'**,**'NBP5'**],** index **=** Measure_date**)**

df **=** df_main**.**merge**(**NBP_0**,**left_index **=** **True,** right_index**=** **True)**

df**[**'Year'**]** **=** df**.**index**.**year

df**[**'Month'**]** **=** df**.**index**.**month

#########################################################

avg_df_transposed_year **=** df**.**groupby**([**'Year'**]).**mean**()**

#yearly changes from baseline processing

avg_df_transposed_year_minus_from_NBP0**=** avg_df_transposed_year**.**sub**(**avg_df_transposed_year **[**"NBP0"**],**axis **=** 0**)**

# percentage change from baseline

avg_df_transposed_year_change_percent_from_NBP0**=** **(**avg_df_transposed_year_minus_from_NBP0**.**div**(**avg_df_transposed_year **[**"NBP0"**]** **,**axis **=**0**))***100

#################################################

# for boxplot

# per year

df_final_for_boxplot_per_year **=** avg_df_transposed_year_change_percent_from_NBP0**.**drop**(**columns **=** **[**"NBP0" **,** "Month"**])**

**return** df_final_for_boxplot_per_year

########## Final output. An example of Flow plot. The same code is applicable for other nutrients and SS###

# flow 111 for RCP-4.5

#30-40

Flow_111_GFDL45_30_40 **=** plot**(**Flow_111_GFDL45**,** Climate_date**,** 'Simo_Flow'**,** " Flow "**,** "(m3/s)"**,** **int(**3652**),** **int(**7305**))**

#40-50

Flow_111_GFDL45_40_50 **=** plot**(**Flow_111_GFDL45**,** Climate_date**,** 'Simo_Flow'**,** " Flow "**,** "(m3/s)"**,** **int(**7305**),** **int(**10957**))**

#50-60

Flow_111_GFDL45_50_60 **=** plot**(**Flow_111_GFDL45**,** Climate_date**,** 'Simo_Flow'**,** " Flow "**,** "(m3/s)"**,** **int(**10957**),** **int(**14610**))**

#60-70

Flow_111_GFDL45_60_70 **=** plot**(**Flow_111_GFDL45**,** Climate_date**,** 'Simo_Flow'**,** " Flow "**,** "(m3/s)"**,** **int(**14610**),** **int(**18262**))**

# flow 111 for RCP-8.5

#30-40

Flow_111_GFDL85_30_40 **=** plot**(**Flow_111_GFDL85**,** Climate_date**,** 'Simo_Flow'**,** " Flow "**,** "(m3/s)"**,** **int(**3652**),** **int(**7305**))**

#40-50

Flow_111_GFDL85_40_50 **=** plot**(**Flow_111_GFDL85**,** Climate_date**,** 'Simo_Flow'**,** " Flow "**,** "(m3/s)"**,** **int(**7305**),** **int(**10957**))**

#50-60

Flow_111_GFDL85_50_60 **=** plot**(**Flow_111_GFDL85**,** Climate_date**,** 'Simo_Flow'**,** " Flow "**,** "(m3/s)"**,** **int(**10957**),** **int(**14610**))**

#60-70

Flow_111_GFDL85_60_70 **=** plot**(**Flow_111_GFDL85**,** Climate_date**,** 'Simo_Flow'**,** " Flow "**,** "(m3/s)"**,** **int(**14610**),** **int(**18262**))**

**def** combined_figure**(**list_1**,** list_2**,** list_3**,** list_4**,** list_5**,** list_6**,** list_7**,** list_8**,** col_name**):**

list_1 **[**"Baseline"**]** **=**"Biomass+RCP-4.5"

list_2 **[**"Baseline"**]** **=**"Biomass+RCP-8.5"

list_3 **[**"Baseline"**]** **=**"Biomass+RCP-4.5"

list_4 **[**"Baseline"**]** **=**"Biomass+RCP-8.5"

list_5 **[**"Baseline"**]** **=**"Biomass+RCP-4.5"

list_6 **[**"Baseline"**]** **=**"Biomass+RCP-8.5"

list_7 **[**"Baseline"**]** **=**"Biomass+RCP-4.5"

list_8 **[**"Baseline"**]** **=**"Biomass+RCP-8.5"

com_df_45 **=** pd**.**concat**([**list_1**,** list_3**,** list_5**,** list_7**],** axis **=** 0**)**

com_df_85 **=** pd**.**concat**([**list_2**,** list_4**,** list_6**,** list_8**],** axis **=** 0**)**

com_df**=** pd**.**concat**([**com_df_45**,** com_df_85**])**

mod_df **=** pd**.**melt**(**com_df**,**id_vars**=**"Baseline"**)**

sns**.**barplot**(**x **=**"variable"**,** y **=** "value"**,** hue **=**"Baseline"**,** data**=** mod_df**,** ci **=** **None,** palette **=**"muted"**)**

sns**.**despine**(**offset**=**1**,** trim **=True)**

plt**.**legend**(**title **=** ""**,** fontsize **=** 12**)**

plt**.**xlabel**(**''**)**

plt**.**ylabel**(**'Annual Change from NBP0 (%)'**,**fontsize **=** 12**)**

plt**.**legend**(**loc **=** 'upper left'**,** prop**={**'size'**:**10**},** ncol **=** 1**)**

plt**.**savefig **(str(**col_name**)+**' yearly_boxplot.jpg'**,** dpi **=** 300**)**

plt**.**show**()**

plt**.**close **()**

# Combine figure for Flow. The same code is applicable for other nutrients and SS###

combined_figure**(**Flow_111_GFDL45_30_40**,**Flow_111_GFDL85_30_40**,**Flow_111_GFDL45_40_50**,**Flow_111_GFDL85_40_50**,** Flow_111_GFDL45_50_60**,**Flow_111_GFDL85_50_60**,** Flow_111_GFDL45_60_70**,** Flow_111_GFDL85_60_70**,** "Flow"**)**
